# Analysis of Safety Concerns on Herbal Products with Assumed Phytoestrogenic Activity

**DOI:** 10.3390/ph16081137

**Published:** 2023-08-10

**Authors:** A. Marije Tjeerdsma, Florence P. A. M. van Hunsel, Sonja van de Koppel, Corine Ekhart, Annabella Vitalone, Herman J. Woerdenbag

**Affiliations:** 1 Department of Pharmaceutical Technology and Biopharmacy, Groningen Research Institute of Pharmacy (GRIP), University of Groningen, Antonius Deusinglaan 1, 9713 AV Groningen, The Netherlands; marijetjeerdsma@gmail.com; 2 Netherlands Pharmacovigilance Centre Lareb, Goudsbloemvallei 7, 5237 MH ’s-Hertogenbosch, The Netherlands; f.vanhunsel@lareb.nl (F.P.A.M.v.H.); vdkoppel@ziggo.nl (S.v.d.K.); c.ekhart@lareb.nl (C.E.); 3 Department of PharmacoTherapy, -Epidemiology & -Economics, Groningen Research Institute of Pharmacy (GRIP), University of Groningen, Antonius Deusinglaan 1, 9713 AV Groningen, The Netherlands; 4 Department of Physiology and Pharmacology ‘Vittorio Erspamer’, Sapienza University of Rome, Piazzale Aldo Moro 5, 00185 Rome, Italy; annabella.vitalone@uniroma1.it

**Keywords:** *Actaea racemosa* L., drug-related side effects and adverse reactions, *Humulus lupulus* L., Netherlands Pharmacovigilance Centre Lareb, *Glycine max* (L.) Merr., phytovigilance, phytoestrogens, *Trifolium pretense* L., VigiBase, *Vitex agnus-castus* L., World Health Organization (WHO)

## Abstract

Phytoestrogens (PEs) are plant-based compounds that can interact with estrogen receptors and are mainly used to treat menopausal complaints. However, the safety of products with assumed phytoestrogenic activity is not fully understood. This study aimed to identify plant species with assumed phytoestrogenic activity, review existing literature on their use and safety, and critically evaluate adverse reaction (AR) reports of single-herb, multi-herb, and mixed-multiple products, as submitted to the Netherlands Pharmacovigilance Centre Lareb and to VigiBase of the World Health Organization (WHO). In the Lareb database, the most commonly reported plant species to cause ARs (total of 67 reports) were *Actaea racemosa* L. (black cohosh) (47.8%), *Humulus lupulus* L. (hops) (32.8%), and *Glycine max* (L.) Merr. (soybean) (22.4%). In the VigiBase database (total of 21,944 reports), the top three consisted of *Glycine max* (L.) Merr. (71.4%), *Actaea racemosa* L. (11.6%), and *Vitex agnus-castus* L. (chaste tree) (6.4%). In the scoping review (total of 73 articles), *Actaea racemosa* L. (30.1%), *Glycine max* (L.) Merr. (28.8%), and *Trifolium pratense* L. (13.7%) were the most frequently mentioned plant species. ARs were most frequently reported in the system organ classes “gastrointestinal disorders”, “skin and subcutaneous tissue disorders”, “reproductive system and breast disorders”, and “general disorders and administration site conditions”. Furthermore, from the scoping review, it appeared that the use of products with assumed phytoestrogenic activity was associated with postmenopausal bleeding. It was concluded that, while the potential benefits of products with assumed phytoestrogenic activity have been extensively pursued, the potential occurrence of ARs after using these products is less well understood. This study highlights the need for further investigation and careful monitoring of these products to better understand their effects and ensure the safety and well-being of individuals using them.

## 1. Introduction

Natural health products containing phytoestrogens (PEs) possess a claim to have potential health benefits and are used for a variety of indications such as cancer prevention, osteoporosis, prostate cancer, cardiovascular disease, metabolic syndrome, and menopausal complaints [1]. Moreover, PEs are considered an alternative to hormone replacement therapy (HRT). HRT is commonly prescribed to women experiencing severe symptoms during the menopausal transition and after menopause, including an increased risk of developing osteoporosis, hot flashes, and genital atrophy [2]. Despite the effectiveness of HRT, its use has been linked to an elevated risk of breast cancer with increasing total duration of use and endometrial cancer even when treatment lasts less than five years [3,4]. To avoid such adverse effects caused by HRT, natural products containing PEs receive considerable interest from the public. However, the question arises whether PEs are really sufficiently safe to use.

**Figure 1:**
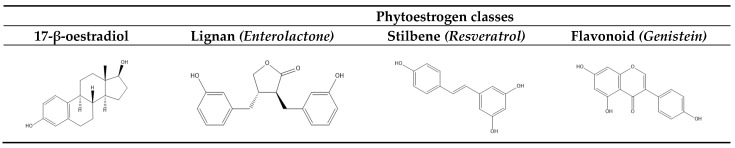
Structural formulas of the endogenous estrogen hormone 17-β-estradiol and representatives of PEs from the main classes.

PEs represent a diverse group of plant-derived compounds that are structurally similar to the main endogenous estrogen hormone in women, 17-β-estradiol. PEs are abundant in various foods such as nuts, seeds, fruits, and vegetables and in a number of medicinal plants. PEs are divided into three main classes: lignans, stilbenes and flavonoids, with a subdivision into a number of subclasses [5]. Most PEs belong to the (sub)classes of isoflavones, lignans, or coumestans [6]. The structural similarities with 17-β-estradiol allow PEs to bind to estrogen receptors, displaying a lower affinity than 17-β-estradiol due to their smaller size and less polar nature. Figure 1 shows the similarity in chemical structure between 17-β-estradiol and representatives of the main classes of PEs.

PEs interact with both estrogen receptor alpha (ERα) and estrogen receptor beta (ERβ) by binding to the ligand-dependent AF-2 receptor domain, activating cellular mechanisms mediated via these receptors that can result in either estrogenic or anti-estrogen effects [7,8,9,10,11]. Subtle changes in the structure of PEs can significantly impact their biological activity and binding affinity for estrogen receptors, particularly the number and position of hydroxyl substituents in the chemical structure [12]. It is important to note that PEs can have binding affinities up to 10,000-fold lower than 17-β-estradiol yet still exhibit relatively strong agonistic activity toward estrogen receptors [13]. PEs may also exhibit characteristics of selective estrogen receptor modulators (SERMS) and can have varying agonistic or antagonistic effects depending on the subtype of estrogen receptor they interact with. ERα and ERβ have distinct distributions in tissues and possess different affinities for the various ligands, which allow them to modulate various physiological processes through the regulation of target gene transcription [14]. This also pinpoints the potential for PEs to cause harmful effects.

It is important to note that the bioactivity of plant species may not be exclusively attributed to their PE active constituents, as they may also contain non-PE bioactive constituents that could play a significant role in the mechanism of action for the treatment of menopausal complaints and related indications. Examples of this are *Actaea racemosa* L. (black cohosh), which contains triterpene glycosides as its primary bioactive constituent, and *Vitex agnus-castus* L. (chaste tree), which contains diterpenoids as one of its main components [15,16].

**Figure 2:**
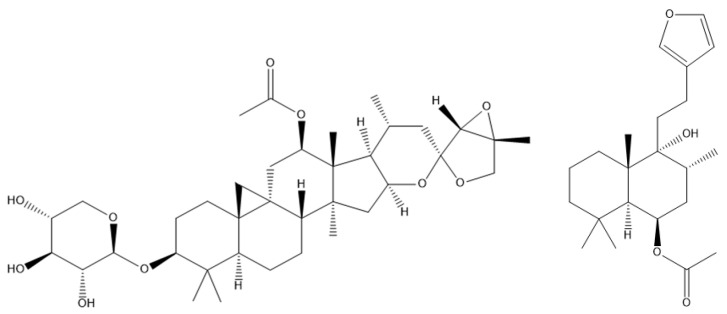
Structural formulas of the triterpene glycoside 23-epi-26-deoxyactein from black cohosh (**left**) and the diterpenoid rotundifuran from chaste tree (**right**).

Triterpene glycosides found in black cohosh comprise a diverse group of chemicals featuring a five-ring structure with varying substitutions. Among these, 23-epi-26-deoxyactein, actein, and cimiracemoside A are most commonly used in standardized black cohosh extracts (Figure 2) [17]. While black cohosh has been suggested to exert direct estrogenic activity, triterpene glycosides have been shown to not bind to estrogenic receptors and therefore do not possess an estrogenic effect [15,18]. In the literature, there is controversy about the phytoestrogenic activity of black cohosh. Rather, the hypothesized mechanism of action of triterpene glycosides involves their interaction with the hypothalamus in a serotonergic manner, leading to the potential alleviation of postmenopausal hot flushes [15].

Although flavonoids with assumed phytoestrogenic activity in chaste tree have been used in the symptomatic treatment of menopausal complaints, diterpenoids are considered to be the major biologically active component of this plant species (Figure 2). Diterpenoids constitute a significant portion of the chaste tree’s total dopaminergic activity and have the ability to interact with dopamine receptors in the brain [19]. Such dopaminergic effects have the potential to alleviate hot flashes, which may prove beneficial in ameliorating the emotional symptoms of menopause [20].

The aim of our study was twofold. First, a scoping review was performed to obtain a complete picture of all plant species with assumed phytoestrogenic activity and to critically review the literature concerning the use and safety of products with assumed phytoestrogenic activity. Second, safety was assessed by analyzing individual case safety reports (ICSRs) that were associated with the use of products with assumed phytoestrogenic activity and were included in VigiBase and in the database of the Netherlands Pharmacovigilance Centre Lareb. VigiBase is the World Health Organization’s (WHO) global database of reported adverse reactions to medicinal products, developed and maintained by the Uppsala Monitoring Centre (UMC) [21]. The findings are discussed in relation to the literature. The results of this study should provide more insight into the safe and responsible use of products with assumed phytoestrogenic activity and highlight possible risks associated with their use.

## 2. Results

### 2.1. Scoping Review

**Figure 3:**
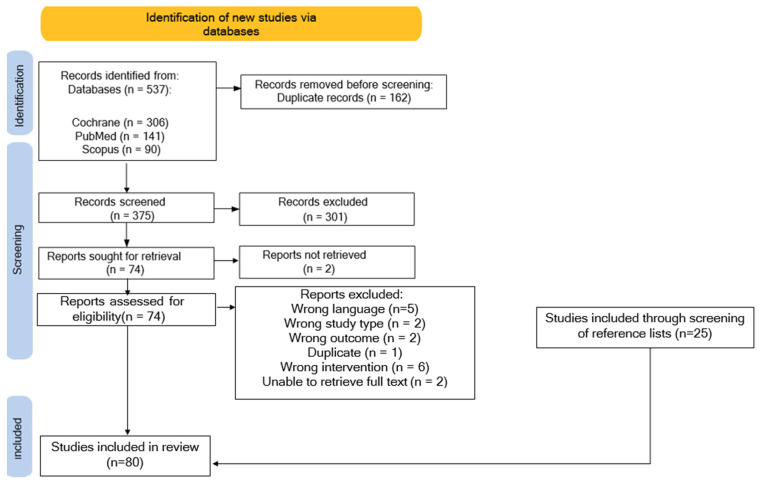
PRISMA flow diagram of included articles in the scoping review.

The scoping review encompassed a total of 80 eligible studies (Figure 3), a total of 82 herbal products were subject to investigation. Of these, 56 products concerned single-herbs and 26 multi-herb products. Table 1 shows the sex and age distribution of participants included in these studies. Table 2 gives an overview of the most frequently used plants. Of the 80 articles, 55 reported on ARs occurring during the study period, in which a total of 1152 ARs were reported. Of those, 950 (82.5%) ARs were associated with single-herb products, and 202 (17.5%) ARs occurred after using multi-herb products.

#### 2.1.1. Single-Herb Products

The scoping review revealed that single-herb products were mainly used by women in the age group of 41–60 (Table 1). A total of 56 single-herb products, derived from 9 different plant species, were used in clinical studies (Table 2). The three most frequently reported plant species were *Actaea racemosa* L. (synonym *Cimicifuga racemosa* (L.) Nutt.) (n = 13), *Glycine max* (L.) Merr. (n = 8), and *Trifolium pratense* L. (n = 7).

All reported ARs in the articles concerning single-herb products were categorized into their respective System Organ Class (SOC) and Preferred Term (PT). For single-herb products, the most commonly found ARs were classified in the SOCs “investigations” (n = 303), followed by “reproductive system and breast disorders” (n = 263), “gastrointestinal disorders” (n = 134), “nervous system disorders” (n = 54), “musculoskeletal and connective tissue disorders” (n = 50), and “infections and infestations” (n = 44) (see Table 3). None of the included articles reported any serious ARs. For the full data we refer to Table S1 (Supplementary Materials).

#### 2.1.2. Multi-Herb Products

The scoping review revealed that single-herb products were mainly used by women in the age group of 41–70 (Table 1). A total of 26 multi-herb products (Table 2), amounting to a total of 126 plants, were used in clinical studies. The most frequently reported plant species were *Glycine max* (L.) Merr. (n = 13) and *Actaea racemosa* L. (synonym *Cimicifuga racemosa* (L.) Nutt (n = 9). Combinations of herbs with the most frequently reported ARs occurring were *Actaea racemosa* L. with *Glycyrrhiza glabra* L. and *Actaea racemosa* L. with *Angelica sinensis* (Oliv.) Diels.

All reported ARs in articles concerning multi-herb products were categorized into their respective SOCs and PTs. The most commonly reported ARs were classified in the SOC “reproductive system and breast disorders” (n = 60), “gastrointestinal disorders” (n = 59), “respiratory, thoracic and mediastinal disorders” (n = 19), “nervous system disorders” (n = 16), “musculoskeletal and connective tissue disorders” (n = 10), and “general disorders” (n = 8) (See Table 4). For the full data, we refer to Table S2 (Supplementary Materials).

### 2.2. Assessment of Reported ARs to the Netherlands Pharmacovigilance Center Lareb

#### 2.2.1. Single-Herb Products

The Netherlands Pharmacovigilance Centre Lareb received a total of 29 reports of suspected ARs associated with the use of single-herb products in the period between 1999 and 2022. The age and sex distributions of these cases are displayed in Table 5. The 29 reports concerning single-herb products contained a total of 6 different plant species. With the three most frequently reported plant species being *Actaea racemosa* L., or synonymously, *Cimicifuga racemosa* L. (n = 16), *Glycine max* (L.) Merr. (n = 3), and *Vitex agnus-castus* L. (n = 3). Table 6 shows an overview of identified plant species.

In 20 of the 29 reports, either only the single-herb product was used at the time of AR reporting or it was the only suspected causative agent of the AR. The most commonly reported SOC of the ARs in these reports were gastrointestinal disorders (n = 10), skin and subcutaneous tissue disorders (n = 7), reproductive system and breast disorders (n = 6), investigations (n = 5), psychiatric disorders (n = 4), and hepatobiliary disorders (n = 3) (see Table 7).

Of these 20 reports, two were classified as serious. One report described a case of autoimmune hepatitis following the oral administration of a product containing *Cimicifuga racemosa* L. The second report documented a life-threatening case of petechiae, hematoma, dizziness, menorrhagia, red discoloration of urine, and swelling under the knee following the oral administration of a product containing *Trifolium pratense* L. As an immediate follow-up on these serious ARs, the product was withdrawn, and the patients were still in the process of recovery at the time of reporting.

In four reports, another product was used concomitantly alongside a single-herb product, which was also classified as being suspected to have caused the AR in question. The most commonly reported SOCs that were involved in the ARs were nervous system disorders (n = 4), general disorders (n = 2), eye disorders (n = 1), and cardiac disorders (n = 1) (see Table 8).

Of these reports, one was classified as serious. This report documented a case of anaphylaxis following the oral administration of a product containing *Cimicifuga racemosa* L., in addition to the concomitant use of laxatives and bisphosphonates. All suspected products were immediately withdrawn; however, the patient’s outcome was unknown.

The remaining five reports involved the use of products primarily advertised to contain minerals and vitamins but also containing a single herb with assumed phytoestrogenic activity. The most commonly reported SOCs that were involved in these ARs were nervous system disorders (n = 4) and investigations (n = 2) (see Table 7). None of these reports were classified as serious.

#### 2.2.2. Multi-Herb Products

The Netherlands Pharmacovigilance Centre Lareb received a total of 38 reports of suspected ARs associated with the use of multi-herb products in the period between 1999 and 2022. The age and sex distributions of these reports are displayed in Table 5. The 38 reports involving multi-herb products contained a total of 30 different plant species, amounting to 167 plants. The three most frequently reported plant species were *Humulus lupulus* L. (n = 22), *Actaea racemosa* L., or synonymously, *Cimicifuga racemosa* L. (n = 16), and *Glycine max* (L) Merr. (n = 12). Table 6 shows an overview of identified plant species.

In 34 of these reports, only multi-herb products were used at the time of reporting the AR, or it was the only suspected product to cause the AR amongst other concomitant medications. The most commonly reported SOCs that were involved in the ARs were reproductive system and breast disorders (n = 10), gastrointestinal disorders (n = 9), nervous system disorders (n = 5), investigations (n = 2), and cardiac disorders (n = 2) (see Table 9).

Out of these 34 reports, three were classified as serious. One report described a case of endometrial hypertrophy and postmenopausal bleeding following the oral administration of a multi-herb product containing *Glycine max* and *Humulus lupulus* L. The second report documented a life-threatening case of acute liver failure and liver transplant following the oral administration of a multi-herb product containing *Cimicifuga racemosa* L., *Salvia officinalis* L., and *Valeriana officinalis* L. The final serious report concerned a case of endometrial hyperplasia, abdominal cramps, and vaginal bleeding following the oral administration of a multi-herb product containing *Humulus lupulus* L. and various types of grain. In all three reports of serious ARs, the product was withdrawn and the patients were in the process of recovery.

In three reports, another product was used concomitantly with a multi-herb product, which was also classified as being suspected to have caused an AR, or multiple multi-herb products were used. The most commonly reported SOCs involved in the ARs were nervous system disorders (n = 3), general disorders and administration site conditions (n = 3), and endocrine disorders (n = 2) (see Table 10).

One of the three reports was classified as serious, involving a life-threatening case of hepatitis and pruritus following the oral administration of three different multi-herb products containing a variety of herbs, including *Cimicifuga racemosa* L., *Linum usitatissimum* L., *Humulus lupulus* L., and *Vitex agnus-castus* L. All multi-herb products were withdrawn, and the patient eventually recovered.

The remaining report involved the use of a product primarily advertised to contain minerals and vitamins but also containing multiple herbs. The SOC involved in this AR was nervous system disorders (n = 1) (See Table 9). This report was considered non-serious.

### 2.3. Assessment of Reported ARs to the WHO-UMC

The WHO received a total of 21,944 individual reports of ICSRs occurring after the use of products with assumed phytoestrogenic activity from plant species as identified in the scoping review. Of these, 4446 were single-herb, 4655 were multi-herb, and 12,843 were mixed-multiple products. The sex and age distributions of these reports are displayed in Table 11. Table 12 shows an overview of all reports concerning the requested plant species submitted to the WHO. The plant species with the most reports were *Glycine max* (L.) Merr. (n = 15,669), followed by *Actaea racemosa* L. (n = 2549) and *Vitex agnus-castus* L. (n = 1401).

#### 2.3.1. Single-Herb Products

A total of 4446 reports from the WHO-UMC regarding single-herb products were analyzed. The Asia-Pacific region submitted the largest number of reports (n = 1889). In 3592 of these reports, only single-herb products with assumed phytoestrogenic activity and no other suspected causative agent were used, or it was the only product used at the time of the AR occurring. The most frequently reported SOCs associated with these reports were “gastrointestinal disorders” (n = 1310), “skin and subcutaneous tissue disorders” (n = 1173), and “general disorders and administration site conditions” (n = 768). Table S3 (Supplementary Materials) displays the most frequently reported SOCs with their respective PTs per plant species.

The remaining 854 reports involved the concomitant use of other products suspected to have been responsible for the ARs, along with single-herb product use. The three most frequently reported SOCs associated with these reports were “general disorders and administration site conditions” (n = 312), “gastrointestinal disorders” (n = 269), and “nervous system disorders” (n = 224). Table S4 (Supplementary Materials) displays the most frequently reported SOCs with their respective PTs.

Out of the 4446 individual case reports associated with single-herb products, 671 were classified as serious, with 337 of them involving the use of only single-herb products and the remaining 334 concerning the concomitant use of other suspected products alongside single-herb products. Of the 337 reports concerning only single-herb products, the most frequently reported serious AR was pruritus (n = 28), followed by rash (n = 21) and nausea (n = 17).

#### 2.3.2. Multiple-Herb Products

The details involving the reports from the WHO-UMC regarding multi-herb products contained a total of 4655 reports, with the Asia-Pacific region having submitted the largest number of reports (n = 2926). In 3097 of these reports, only multi-herb products with assumed phytoestrogenic activity and no other suspected causative agent were used, or it was the only product used at the time of the AR occurring. The most frequently reported SOCs associated with these reports were “gastrointestinal disorders” (n = 1647), “skin and subcutaneous tissue disorders” (n = 647), and “general disorders and administration site conditions” (n = 600) (see Table S5).

The remaining 1558 reports involved the concomitant use of other products suspected to have been responsible for the ARs, along with multi-herb product use. The most frequently reported SOCs associated with these reports were “gastrointestinal disorders” (n = 638), “skin and subcutaneous tissue disorders” (n = 384), and “general disorders and administration site conditions” (n = 347) (see Table S6).

Out of the 4655 individual case reports associated with multi-herb products, 578 were classified as serious, with 252 of them involving the use of only multi-herb products and the remaining 326 concerning the concomitant use of other suspected products alongside multi-herb products. Of the 252 reports concerning only multi-herb products, the most frequently reported serious AR was nausea (n = 19), followed by dizziness (n = 16) and diarrhea (n = 14).

#### 2.3.3. Mixed-Multiple Products

A total of 12,843 reports from the WHO-UMC regarding mixed-multiple products were analyzed. The Asia-Pacific region submitted the largest number of reports (n = 9389). In 10,140 of these reports, only mixed-multiple products with assumed phytoestrogenic activity and no other suspected causative agent were used, or it was the only product used at the time of the AR occurring. The most frequently reported SOCs associated with these reports were “general disorders and administration site conditions” (n = 5173), “gastrointestinal disorders” (n = 3424), and “skin and subcutaneous tissue disorders” (n = 2209) (See Table S7).

The remaining 2703 reports involved the concomitant use of other products suspected to have been responsible for the ARs, along with mixed-multiple product use. The most frequently reported SOCs involved were “general disorders and administration site conditions” (n = 1799), “gastrointestinal disorders” (n = 1465), and “skin and subcutaneous tissue disorders” (n = 561) (see Table S8).

Out of the 12,843 individual case reports associated with mixed-multiple products, 3549 were classified as serious, with 2279 of them involving the use of only mixed-multiple products and the remaining 1270 concerning the concomitant use of other suspected products alongside mixed-multiple products. Of the 2279 reports concerning only mixed-multiple products, the most frequently reported serious AR was chills (n = 745), followed by pyrexia (n = 421) and hyperpyrexia (n = 352).

## 3. Discussion

The use of herbal products with assumed phytoestrogenic activity is receiving considerable interest due to their potential benefits in managing menopausal complaints. However, concerns have been raised regarding their safety, especially with regards to ARs that may occur. The present study provides a comprehensive overview of the current scientific status of the safety of products with assumed phytoestrogenic activity based on a scoping review covering the period from January 2000 to October 2022. Additionally, AR reports associated with the use of products with assumed phytoestrogenic activity were thoroughly assessed. The AR reports were obtained from the nationwide spontaneous reporting database of the Netherlands Pharmacovigilance Center Lareb and from the global database Vigibase of the WHO-UMC.

The scoping review identified *Actaea racemosa* L. (black cohosh) and *Glycine max* (L.) Merr. (soybean) as the most commonly reported plant species to cause ARs in single-herb products with assumed phytoestrogenic activity. *Vitex agnus*-castus L. (chaste tree) and *Trifolium pratense* L. (red clover) also received a considerable number of reports. These findings are consistent with the Lareb and WHO databases, which also report a high number of ARs associated with these plant species. The high number of reports regarding these plant species may be attributed to their popularity and their extensive use over time, given that black cohosh preparations have been available at least in Germany since 1956 [22,23,24].

Black cohosh is known to contain triterpene glycosides, such as actein and cimiracemoside A, which act as selective serotonin reuptake inhibitors (SSRIs) and interact with the hypothalamus in a serotonergic manner [15,17]. Although triterpene glycosides are not estrogenic, black cohosh has been suggested to exert tissue-specific action, acting as an estrogen agonist in certain tissues and an estrogen antagonist in others [15,18]. Early studies attributed the effect of black cohosh to the estrogenic isoflavone formononetin, although this finding was not supported by subsequent studies [25]. The constituents responsible for the estrogenic effect in black cohosh remain unclear, and the discussion remains whether the isoflavone formononetin is present in black cohosh [26]. The multi-action mechanism of action of black cohosh suggests its potential for ARs to occur in various systems and tissues.

Soybean and red clover share similar bioactive constituents, such as the isoflavones daidzein, genistein, and glycitein [27,28]. The binding to estrogenic receptors and transactivation capacities differ, with genistein exhibiting the highest relative binding affinity, followed by daidzein [13]. Both isoflavones display transactivation activities between 72 and 85% compared to 17-β-estradiol, where transactivation activity is defined as the stimulation of transcriptional activity mediated by estrogen receptor alpha and beta [29]. At high concentrations, genistein has been found to be more potent than 17-β-estradiol and may give rise to a higher risk of ARs occurring [13]. Red clover predominantly contains formononetin and biochanin A, with lower concentrations of genistein and daidzein [30]. Formononetin and biochanin A have been found to have low binding capacities to estrogen receptors, which could be a possible explanation as to why red clover shows fewer ARs compared to soybean in the WHO database, as the more estrogenically potent genistein and daidzein are present in lower concentrations [30].

The chaste tree contains both flavonoids and diterpenes as its main bioactive constituents [16]. Apigenin, a flavone present in chaste tree, is the most active PE and selectively targets estrogen receptor beta [31]. Although it has a relative binding affinity of 6% compared to 17-β-estradiol, its transcriptional activity was found to be relatively strong, reaching 49% [13]. However, chaste tree possesses other mechanisms of action in addition to its estrogenic effect, setting it apart from red clover and soybean. Diterpenes represent the other major constituents of chaste tree and have a dopaminergic effect by stimulating dopamine D2 receptors in the anterior pituitary [19]. Products with chaste tree may also interact with opioid receptors, although the precise mechanism of action remains unknown [32]. Like black cohosh, the chaste tree’s multi-action mechanism of action suggests that ARs may occur in various systems and tissues.

The scoping review and both databases revealed that more women than men reported ARs after the use of products with assumed phytoestrogenic activity. This sex difference may be attributed to the fact that most studies in the scoping review, as well as reports in the Lareb and WHO databases, focused on the use of these products for the management of menopausal complaints, a condition that affects women exclusively. The most common age group for reporting ARs of single-herb products was found to be between 41 and 60 years old, corresponding to the age range for menopause. Aside from managing menopausal complaints, products with assumed phytoestrogenic activity have been suggested for other indications, including the prevention of osteoporosis and cardiovascular disease, as well as breast cancer prevention, a protective role in the development of prostate cancer, and breast enhancement [1,33,34,35]. However, it is important to note that the evidence for these indications is limited and conflicting. While some studies present potential benefits for the use of these indications, others indicate no effects or benefits. Consequently, the use of products with assumed phytoestrogenic activity for these purposes is still controversial.

The composition and form of the products reported in the scoping review and both databases are highly variable due to differences in plant parts used, methods of preparation (powdered plant material, dried extracts prepared with various solvents), possible standardization, dosage, and duration of exposure [36]. This variability in plant material will impact the quality of the final product and may lead to unknown ingredient concentrations. Concomitantly, biological activity, including safety aspects, will differ among the various products available and may be less predictable if the nature and composition are not known or disclosed in the product information. Additionally, factors such as the quality and identity of the source plant material, fluctuations in content, environmental conditions, and inadequate post-harvest handling and storage may compromise product quality and safety [36]. Determining a safe dosage and duration of exposure for products with assumed phytoestrogenic activity is challenging due to significant variations in the usage of products containing the same plant species.

Mislabeling is another issue, as products with assumed phytoestrogenic activity purchased online often lack adequate product information, and product labels may not provide accurate botanical names or information regarding contained ingredients. Standardized products usually offer more complete background information. Mislabeling of herbal products may result in toxic plant species being used, as they are present in these products, probably as adulterations or substitutions. For example, black cohosh has been associated with a number of case reports of hepatotoxicity. The mechanism by which black cohosh might have caused hepatotoxicity has been extensively debated. A study on two case reports demonstrated that the mechanism of liver injury due to black cohosh is idiosyncratic, leading to gradual hepatocellular disappearance [37]. However, it is suspected that some case reports may be due to the presence of an adulterant, specifically a species other than *Actaea racemosa* L. [38]. In a study from 2019, of 36 dietary supplements claimed to contain black cohosh (*Actaea racemosa* L.), nine were found to contain other Asian *Actaea* species (*A. cimicifuga, A. dahurica, and A. simplex*) rather than black cohosh, which may be hepatotoxic to humans [39]. Most reports of hepatotoxicity associated with black cohosh products provide little to no information about the composition of the product, making causality difficult to determine. It rather shows the importance of rigorous quality control than the unsafeness of “real” black cohosh.

Despite the use of Latinized names in botanical nomenclature, it does not inherently guarantee legitimacy or scientific precision. It was found that the pharmaceutical name “Cimicifugae rhizome” was employed by different pharmacopoeias to refer to substances derived from five different plant species, which have differing chemistries and uses [40]. Rigorous quality control, including information on the identity of the plant species used, is of utmost importance to guarantee products that are safe to use by the consumer.

Regarding the reported ARs of single-, multi-, and mixed-multiple products, the three most frequently reported SOCs were “general disorders and administration site conditions”, “gastrointestinal disorders”, and “skin and subcutaneous tissue disorders”. This is not surprising, as these SOCs are frequently reported to occur generally. A large descriptive analysis conducted by Dubrall et al. on spontaneous reports of medicinal products showed that the most frequently reported ARs belonged to the SOCs “gastrointestinal disorders”, followed by “skin and subcutaneous tissues”, “general disorders and administration site disorders”, and “nervous system disorders” [41]. Moreover, the scoping review and the Lareb database reported a high frequency of the SOC “reproductive system and breast disorders”, with the PT “vaginal spotting” being reported frequently for products containing black cohosh. Lareb has issued multiple signals and publications on postmenopausal bleeding as a potential AR linked to long-term use of products with assumed phytoestrogenic activity [42,43]. Although postmenopausal bleeding is a relatively common symptom after the final menstrual period [44], cases reported to Lareb have described patients endometrial proliferation and subsequent vaginal hemorrhage after usage of these products [45,46]. Notably, the scoping review highlighted SOC “investigations” as the most commonly reported category for single-herb products, encompassing PTs such as increased low-density lipoprotein (LDL) (n = 101), increased triglycerides (n = 97), and increased total cholesterol (n = 95) (see Table 3). It should be acknowledged that almost all reports pertaining to the SOC “investigations” originated from a single study within the scoping review. This study conducted by Raus et al. indicated that the increases in total cholesterol and LDL were documented as separate ARs, although they were actually interrelated. Furthermore, it was emphasized that these PTs constituted approximately 60% of potentially/probably related ARs, with no causal relationship established with the study product [47]. Among the serious reports in which products with assumed phytoestrogenic activity were administered orally, general and gastrointestinal disorders were the most common, with reactions such as chills, hyperpyrexia, and nausea occurring.

For multi-herb and mixed-multiple products with assumed phytoestrogenic activity, establishing a causative relationship between ARs and the product with assumed phytoestrogenic activity is more difficult, as other potentially suspected agents such as co-medication and comorbidities cannot be excluded. However, the nature of the ARs was comparable to those reported for single-herb products.

Oral administration is the most common route for products with assumed phytoestrogenic activity. However, alternative routes of administration are currently being developed, including transdermal application. Transdermal drug delivery may be a promising alternative to oral administration, as it seems to ensure a consistent and steady permeation of the product through the skin, resulting in a more stable plasma concentration of the product and potentially reducing the risk of ARs [48,49]. Researchers are currently focusing on the development of transdermal applications using various nano-carriers to overcome challenges associated with phytoestrogen delivery, such as poor biological availability [50].

It is noteworthy that in the WHO reports on soybeans concerning mixed-multiple products, intravenous administration was found to be the most common route of administration, with parenteral nutrition being the most common indication. Soybean oil is one of the fatty components of parenteral nutrition, next to glucose and amino acids. Soybean mixed-multiple products were the only products for which oral administration was not the most common route; this did not have an effect on the outcomes of the most common SOCs and PTs. As previously mentioned, genistein is one of the main isoflavones in soybean [27]. Genistein has poor oral bioavailability due to various factors, including low water solubility, poor absorption, and a significant first-pass metabolism [51]. Intravenous administration of genistein results in higher bioavailability and shows higher peak concentrations in plasma compared to oral administration [52]. Higher peak concentrations may lead to an increased risk of ARs, which may explain the higher number of ARs with intravenous administration of soybean mixed-multiple products compared to oral administration. However, pure soybean oil could have a different composition from the dry extracts of soybean that are given orally. Therefore, a direct comparison is difficult.

This study’s main strengths lie in its combination of a scoping review that specifically focuses on the safety concerns and ARs reported in the literature with an in-depth analysis of ARs collected in national and global databases. This targeted approach highlights knowledge gaps and areas for further research, including the need for a better understanding of the mechanism of action of PEs and investigating the causality of AR reports. This study also has several limitations, such as the use of concomitant medication in a multitude of reports that were not classified as suspect but could have potentially contributed to the development of ARs. Moreover, underreporting is a common issue in spontaneous reporting systems such as Lareb and Vigibase, leading to a distorted and incomplete picture of the incidence and severity of ARs. It was beyond the scope of this research to perform a causality assessment for individual cases. In addition, the researchers did not have access to the full narrative of cases in Vigibase like they did for the Dutch AR reports. Lastly, the quality of the data retrieved from literature and the databases used in this study may be subject to inconsistencies due to missing information on key points such as the use of alternative plant names or precise dosages.

## 4. Materials and Methods

### 4.1. Scoping Review

This scoping review complies with the Preferred Reporting Items for Systematic Reviews and Meta-Analyses (PRISMA) Extensions for Scoping reviews (PRISMA-Scr) guidelines [53]. The scoping review was performed by conducting a search in three global electronic databases: PubMed, the Cochrane Library, and Scopus, with date limitations set between January 2000 and October 2022. Only articles written in the Dutch and English languages were included. The search strategy was limited to the titles and abstracts of the articles. Additional articles were identified through the screening of reference lists of selected publications and included when meeting the proposed inclusion criteria. The scoping review covered the identification of plant species with assumed phytoestrogenic activity and their safety.

The search strategy used was as follows:

1. (Phytoestrogens “[MeSH] OR phytoestrogen* OR “plant (o)estrogen” OR phyto-(o)estrogen OR “plant (o)estrogens” OR phytoestrogenic);
2. (Safety OR safe* OR “adverse reaction” OR “adverse event” OR “side effect” OR “adverse drug reaction” OR adr OR “undesirable effect” OR tolerability OR safe OR “adverse reactions” OR “adverse events” OR “side effects” OR “adverse drug reactions” OR “undesirable effects” OR safety OR risk OR risks);
3. #1 AND #2.

Articles were included or excluded based on predetermined eligibility criteria. Initial screening of titles and abstracts was performed, and articles with uncertainty regarding eligibility were subjected to full-text review.

Studies were included if they met one or more of the following inclusion criteria:

1. Clinical trials, case reports, and randomized controlled trials making use of products with assumed phytoestrogenic activity in the form of an extract or raw herbal material;
2. Reported or monitored outcomes of ARs after use of products with assumed phytoestrogenic activity;
3. Mentioning of plant species with assumed phytoestrogenic activity.

Studies were excluded if they met one or more of the following exclusion criteria:

1. The full text of a paper was not available;
2. The article was a review;
3. The study made use of animal models only;
4. Articles that investigated the use of isolated compounds for intervention.

### 4.2. Data Analysis and Extraction

#### 4.2.1. Scoping Review

Articles were selected, and duplicates were identified and removed using EndNote X9^®^ software [54]. In the initial stage of research, one reviewer independently evaluated titles and abstracts against the predefined inclusion criteria. Subsequently, the studies were categorized as “included”, “maybe included”, or “excluded”. Full-text retrieval was conducted for studies that potentially met the inclusion criteria. Studies that were deemed ineligible based on title or abstract were excluded, and the reasons for exclusion were documented in the research notes. In cases where there were uncertainties regarding the relevance of a study or if the abstract lacked clarity, the complete article was retrieved for assessment. Full-text studies that did not meet the inclusion criteria were excluded, and the reasons for exclusion were documented in the final report. In cases of doubt concerning the eligibility of a study, inclusion or exclusion was determined through agreement amongst all researchers. The search results were presented in a PRISMA flow diagram.

Information was identified and extracted in an extraction tool in MS Excel in relation to the review’s objectives, covering the identification of plant species with assumed phytoestrogenic activity and their safety. Data from the included articles were collected using a charting table specifically designed to align with the objective of this scoping review. This extraction tool was designed based on the JBI template Source of Evidence Details, Characteristics, and Results Extraction Instrument [55]. The results of the scoping review were organized in an Excel table, which included the following elements: author, year of publication, title of the publication, study type, sex, mean age, country, study objective, number of participants, duration of study, type of intervention used, comparator, plant species used (Latin plant names), plant part used, dosage, concomitant use of other products or medication, and adverse reactions reported. One reviewer independently charted the first ten studies using the extraction instrument. Afterward, all researchers convened to evaluate whether the data extraction method was consistent with the research questions and objectives.

#### 4.2.2. Lareb Reports

AR reports submitted to the Netherlands Pharmacovigilance Centre Lareb, which maintains the Dutch spontaneous reporting database, between 1999 and 2022 were collected and analyzed. The reports related to products with assumed phytoestrogenic activity in either a single-herb or a multi-herb formulation and were submitted to Lareb by healthcare professionals (physicians and pharmacists), consumers, or other non-healthcare professionals. Through an SQL query, the set of products with assumed phytoestrogenic activity in either a single-herb or multi-herb product and their reported ARs were extracted from the Lareb database [56]. This was combined with a search in the Lareb database for products containing vitamins and/or minerals in addition to one or more herbal ingredients (mixed-multiple products). For each individual report, the worldwide unique case identification number was extracted, along with (as far as information was available) the year of reporting, the name of the product involved, the type of reporter, the sex and age of the patient or user, the action taken after using the product that caused an AR, a description of the AR that occurred, the reported System Organ Classes (SOCs), the reported Preferred Term (PT), the seriousness of the AR, indication for use of the product, co-medication, the outcome of the event, the ingredients of the product, time to onset, dose, dosage regimen, a summary of the report, and a narrative.

#### 4.2.3. WHO Reports

Data analysis was performed on Individual Case Safety Reports (ICSRs) of ARs in VigiBase, a global database of the World Health Organization (WHO) for reported potential ARs of medicinal products maintained by the Uppsala Monitoring Centre (UMC) since 1978. VigiBase contains over 30 million reports of suspected ARs submitted by member countries of the WHO Program for International Drug Monitoring (PIDM) from over 170 countries and territories, spanning 99% of the world’s population. Case reports of ARs are collected, coded, and assessed in their country of origin.

A custom search was requested with data from a frozen dataset from the VigiBase database on 22 November 2022, which included names of plant species with assumed phytoestrogenic activity, as identified in the scoping review. The search criteria included single-, multi-, and mixed-multiple products, with drug involvement being set to suspected/interacting. The search was performed for all ARs and countries since the start of Vigibase until the date of extraction (22 November 2022). Fields included in the search were case ID, sex and age of the patient, seriousness of the AR, suspect/interacting drug, ingredients of the product, action taken, reaction System Organ Class (SOC) involved, reaction preferred term (PT), outcome event, qualification of the reporter, year of reporting, indication, concomitant medication, time to onset, dose, and dosage regimen.

### 4.3. Categorization of Adverse Reactions (ARs)

The ARs reported in the reports submitted to Lareb, the WHO, and in the scoping review were coded using the Medical Dictionary for Regulatory Activities (MedDRA). MedDRA is a medical terminology developed by the International Council for Harmonization of Technical Requirements for Pharmaceuticals for Human Use and is used to classify adverse event data from spontaneous adverse event reports in a standardized and hierarchal manner [57]. The reported ARs were categorized according to their SOCs and the most commonly reported PTs.

Reports of a serious AR, defined as “one which requires hospitalization or prolongation of existing hospitalization, causes congenital malformation, results in persistent or significant disability or incapacity, is life-threatening, or results in death”, were evaluated in more detail [58].

Descriptive statistics were used in Microsoft Excel 2019 for analyzing the Lareb and WHO reports, and the data was presented using bar graphs.

### 4.4. Categorization of the Results of Reports from Lareb and the WHO

The following four categories were used to group the results:

1. Reports with a single-herb product being the only used product at the time the AR was reported, or it was the only suspected product amongst other concomitant medication;
2. Reports of which other products were concomitantly used with a single-herb product were also classified as being suspect of causing the AR;
3. Reports with a multi-herb product being the only used product at the time the AR was reported, or it was the only suspected product amongst other concomitant medications;
4. Reports of other products that were concomitantly used with a multi-herb product were also classified as being suspect in causing the AR.

## 5. Conclusions

While the possible beneficial effects of PE consumption have been extensively pursued, the potential for causing ARs has received less attention. Our study is an extensive safety evaluation based on a scoping review and analysis of pharmacovigilance databases from Lareb and WHO and presents an overview of all plant species with assumed phytoestrogenic activity and their potential to cause ARs. *Actaea racemosa* L. (black cohosh), *Glycine max* (L.) Merr. (soybean), *Vitex agnus-castus* L. (chaste tree), and *Trifolium pratense* L. (red clover) were the most commonly reported plant species to cause ARs. The most frequently reported ARs were mild and included nausea, pruritus, and pyrexia. It is noteworthy that products with assumed phytoestrogenic activity have been associated with postmenopausal bleeding in the scoping review. Further investigation and careful monitoring of these products are warranted to better understand their effects and ensure the safety and well-being of individuals using them.

## Figures and Tables

**Table 1:** Sex and age distribution of participants in clinical studies using single- and multi-herb products from the scoping review.

Age Group	Single-Herb Products (n=) (See Section 2.1.1)	Multi-Herb Products (n=) (See Section 2.1.2)	Total (n=) and % *
0–10	0	0	0 (0.0%)
11–20	0	0	0 (0.0%)
21–30	1	0	1 (1.4%)
31–40	2	1	3 (4.1%)
41–50	6	3	9 (12.3%)
51–60	38	14	52 (71.2%)
61–70	0	2	2 (2.7%)
71–80	0	0	0 (0.0%)
81–90	0	0	0 (0.0%)
91–100	0	0	0 (0.0%)
Unknown	5	1	6 (8.2%)
**Sex**			
Male	0	0	0 (0.0%)
Female	51	19	70 (95.8%)
Unknown	1	2	3 (4.1%)

* Calculated as the percentage of the total of included articles excluding case reports (n = 73).

**Table 2:** The most frequently reported plant species in single- and multi-herb products from the scoping review.

Plant Species	Single-Herb Products Times Reported (n=) (See Section 2.1.1)	Multi-Herb Products Times Reported (n=) (See Section 2.1.2)	Total (n=) and % *
*Actaea racemosa* L.	13	9	22 (30.1%)
*Glycine max* (L.) Merr.	8	13	21 (28.8%)
*Trifolium pratense* L.	7	3	10 (13.7%)
*Humulus lupulus* L.	4	3	7 (9.6%)
*Linum usitatissimum* L.	1	5	6 (8.2%)
*Vitex agnus-castus* L.	-	5	6 (8.2%)
*Angelica sinensis* (Oliv.) Diels	-	5	5 (6.8%)
*Trigonella foenum-graecum* L.	5	-	5 (6.8%)
*Glycyrrhiza glabra* L.	-	5	5 (6.8%)
*Pueraria mirifica* Airy Shaw and Suvat.	4	-	4 (5.5%)
*Cornus officinalis* Siebold and Zucc.	-	3	3 (4.1%)
*Rehmannia glutinosa* (Gaertn.) DC.	-	3	3 (4.1%)
*Astragalus membranaceus* Fisch. Ex Bunge	-	3	3 (4.1%)
*Foeniculum vulgare* Mill.	2	1	3 (4.1%)
*Panax ginseng* C.A. Mey	2	1	3 (4.1%)

* Calculated as the percentage of the total of included articles excluding case reports (n = 73).

**Table 3:** Most frequently reported ARs in single-herb products from the scoping review categorized by System Organ Class (SOC) and the most frequently reported Preferred Terms (PTs).

System Organ Class (SOC)	Times Reported (n=)	Percentage (%) *	Preferred Term (PT)	Times Reported (n=)	Percentage (%) *
Investigations	303	31.9	Increased LDL	101	10.6
			Increased triglycerides	97	10.2
			Increased total cholesterol	95	10.0
Reproductive system and breast disorders	263	27.7	Vaginal bleeding/spotting	131	13.8
			Breast pain/tenderness	71	7.5
			Proliferative/abnormal endometrium	19	2.0
Gastrointestinal disorders	134	14.1	Gastrointestinal complaints (unspecified)	68	7.2
			Nausea and vomiting	22	2.3
			Abdominal discomfort/pain	13	1.4
Nervous system disorders	54	5.7	Headache	28	2.9
			Dizziness	12	1.3
			Nervous system disorders (unspecified)	9	0.9
Musculoskeletal and connective tissue disorders	50	5.3	Myalgia	18	1.9
			Musculoskeletal and connective tissue disorders (unspecified)	15	1.6
			Arthralgia	12	1.3
Infections and infestations	44	4.6	Cold or upper respiratory tract infection	20	2.1
			Infections and infestations (unspecified)	13	1.4
			Cystitis	8	0.8

* Calculated as the percentage of the total ARs reported during use of single-herb products (n = 950).

**Table 4:** Reported AR in multi-herb products from the scoping review categorized by System Organ Class (SOC), and the most frequently reported Preferred Terms (PTs).

System Organ Class (SOC)	Times Reported (n=)	Percentage (%) *	Preferred Term (PT)	Times Reported (n=)	Percentage (%) *
Reproductive system and breast disorders	60	29.7	Vaginal bleeding/spotting	28	13.9
			Thickened endometrium	21	10.4
			Breast pain/tenderness	8	4.0
Gastrointestinal disorders	59	29.2	Diarrhea/loose stools	17	8.4
			Gastrointestinal disorders (unspecified)	10	5.0
			Nausea	9	4.5
Respiratory, thoracic, and mediastinal disorders	19	9.4	Respiratory, thoracic, and mediastinal disorders (unspecified)	18	8.9
			Aggravation of asthma	1	0.5
Nervous system disorders	16	7.9	Nervous system disorders (unspecified)	13	6.4
			Headache	3	1.5
Musculoskeletal and connective tissue disorders	10	5.0	Musculoskeletal and connective tissue disorders (unspecified)	10	5.0
General disorders	8	4.0	General disorders (unspecified)	6	3.0
			Oedema	1	0.5
			Influenza	1	0.5

* Calculated as the percentage of the total AR reported during use of multi-herb products (n = 202).

**Table 5:** Sex and age distribution of single- and multi-herb products from reports submitted to the Netherlands Pharmacovigilance Center Lareb.

Age Group	Single-Herb Products (n=) (See Section 2.2.1)	Multi-Herb Products (n=) (See Section 2.2.2)	Total (n=) and % *
0–10	0	0	0 (0.0%)
11–20	0	0	0 (0.0%)
21–30	1	0	1 (1.5%)
31–40	2	1	3 (4.5%)
41–50	8	12	20 (29.9%)
51–60	16	21	37 (55.2%)
61–70	1	4	5 (7.5%)
71–80	0	0	0 (0.0%)
81–90	0	0	0 (0.0%)
91–100	0	0	0 (0.0%)
Unknown	1	0	1 (1.5%)
**Sex**			
Male	0	0	0 (0.0%)
Female	29	38	67 (100.0%)
Unknown	0	0	0 (0.0%)

* Calculated as the percentage of the total reports (n = 67).

**Table 6:** The most frequently reported plant species in single- and multi-herb products from reports submitted to the Netherlands Pharmacovigilance Center Lareb.

Plant Species	Single-Herb Products Times Reported (n=) (See Section 2.2.1)	Multi-Herb Products Times Reported (n=) (See Section 2.2.2)	Total (n=) and % *
*Actaea racemosa* L.	16	16	32 (47.8%)
*Humulus lupulus* L.	-	22	22 (32.8%)
*Glycine max* (L.) Merr.	3	12	15 (22.4%)
*Zea mays* L.	-	10	10 (14.9%)
*Triticum aestivum* L.	-	10	10 (14.9%)
*Secale cereale* L.	-	10	10 (14.9%)
*Hordeum vulgare* L.	-	10	10 (14.9%)
*Fagopyrum esculentum* Moench	-	10	10 (14.9%)
*Avena sativa* L.	-	10	10 (14.9%)
Semen hordei vulgaris germinatum	-	10	10 (14.9%)
*Vitex agnus-castus* L.	3	5	8 (11.9%)
*Capsicum annuum* L.	-	8	8 (11.9%)
*Prunus cerasus* Scop.	-	7	7 (10.4%)
*Dioscorea villosa* L.	-	4	4 (6.0%)
*Lepidium meyenii* Walp.	1	3	4 (6.0%)
*Angelica sinensis* (Oliv.)	3	1	4 (6.0%)
*Trifolium pratense* L.	3	-	3 (4.5%)
*Salvia officinalis* L.	-	3	3 (4.5%)

* Calculated as the percentage of the total reports (n = 67).

**Table 7:** Data on reports submitted to the Netherlands Pharmacovigilance Centre Lareb in which only single-herb products were used at the time the AR was reported or it was the only suspected product to cause the AR amongst other concomitant medications categorized by System Organ Class (SOC) and the most commonly reported Preferred Terms (PTs).

System Organ Class (SOC)	Times Reported (n=)	Preferred Term (PT)	Times Reported (n=)
*Single-Herb Products*			
Gastrointestinal disorders	10	Abdominal pain/discomfort	3
		Nausea	3
		Diarrhea	2
		Vomiting	2
Skin and subcutaneous tissue disorders	7	Rash	2
		Pruritus	2
		Eczema	1
		Petechiae	1
		Skin exfoliation	1
Reproductive system and breast disorders	6	Postmenopausal hemorrhage	2
		Vaginal hemorrhage	1
		Breast pain	1
		Heavy menstrual bleeding	1
		Polymenorrhoea	1
Investigations	5	Weight increased	1
		ALAT increased	1
		GGT increased	1
		Hepatic enzymes increased	1
		Liver function test increased	1
Psychiatric disorders	4	Depressed mood	2
		Anxiety	1
		Suicidal ideation	1
Hepatobiliary disorders	3	Hepatic function abnormal	2
		Autoimmune hepatitis	1
Musculoskeletal and connective tissue disorders	2	Myalgia	1
		Back pain	1
Nervous system disorders	2	Dizziness	1
		Migraine	1
General disorders	2	Swelling	1
		Malaise	1
Vascular disorders	1	Hematoma	1
Renal and urinary disorders	1	Chromaturia	1
Respiratory, thoracic, and mediastinal disorders	1	Respiratory disorder	1
Metabolism and nutrition disorders	1	Decreased appetite	1
*Single-Herb Products Consisting Mainly of Vitamins and Minerals*			
Nervous system disorders	4	Fatigue	1
		Paraesthesia	1
		Hypoesthesia	1
		Neuralgia	1
Investigations	2	Vitamin B6 increased	2
Musculoskeletal and connective tissue disorders	2	Muscle spasms	2
Metabolism and nutrition disorders	1	Decreased appetite	1
Gastrointestinal disorders	1	Nausea	1

**Table 8:** Data on reports submitted to the Netherlands Pharmacovigilance Centre Lareb of which other products were concomitantly used with a single-herb product that has also been classified as being suspect to cause an AR, categorized by System Organ Class (SOC) and the most commonly reported Preferred Terms (PTs).

System Organ Class (SOC)	Times Reported (n=)	Preferred Term (PT)	Times Reported (n=)
Nervous system disorders	4	Loss of consciousness	1
		Peroneal nerve palsy	1
		Paraesthesia	1
		Memory impairment	1
General disorders	2	Fatigue	1
		Chest pain	1
Cardiac disorders	1	Arrhythmia	1
Immune system disorder	1	Anaphylaxis	1
Eye disorders	1	Ocular discomfort	1

**Table 9:** Data on reports submitted to the Netherlands Pharmacovigilance Center Lareb in which only multi-herb products were used at the time the AR was reported or it was the only suspected product to cause the AR amongst concomitant medication, categorized by System Organ Class (SOC) and the most commonly reported Preferred Terms (PTs).

System Organ Class (SOC)	Times Reported (n=)	Preferred Term (PT)	Times Reported (n=)
*Multi-Herb Products*			
Reproductive system and breast disorders	25	Postmenopausal hemorrhage	10
		Vaginal hemorrhage	6
		Endometrial hyperplasia	6
		Endometriosis	1
		Vaginal discharge	1
		Breast pain	1
Gastrointestinal disorders	12	Abdominal discomfort/pain	7
		Constipation	1
		Eructation	1
		Esophageal pain	1
		Dyspepsia	1
		Dry mouth	1
Nervous system disorders	5	Dysgeusia	1
		Polyneuropathy	1
		Headache	1
		Neuropathy peripheral	1
		Paraesthesia	1
Investigations	2	Vitamin B6 increased	2
Cardiac disorders	2	Arrhythmia supraventricular	1
		Sinus tachycardia	1
Hepatobiliary disorders	1	Acute hepatic failure	1
Surgical and medical procedures	1	Liver transplant	1
Skin and subcutaneous tissue disorders	1	Rash maculo-papular	1
*Multi-Herb Products Consisting Mainly of Vitamins and Minerals*			
Nervous system disorders	1	Dizziness	1

**Table 10:** Data on reports submitted to the Netherlands Pharmacovigilance Center Lareb of which other products were concomitantly used with a multi-herb product that has also been classified as being suspect to cause an AR, categorized by SOC and the most commonly reported preferred terms (PTs).

System Organ Class (SOC)	Times Reported (n=)	Preferred Term (PT)	Times Reported (n=)
General disorders and administration site conditions	4	Drug interaction	4
Nervous system disorders	3	Neuropathy peripheral	1
		Memory impairment	1
		Headache	1
Endocrine disorders	2	Goitre	1
		Hypothyroidism	1
Psychiatric disorders	1	Depression	1
Hepatobiliary disorders	1	Hepatitis	1
Skin and subcutaneous tissue disorders	1	Pruritus	1

**Table 11:** Sex and age distribution of single- and multi-herb and mixed-multiple products from reports submitted to the WHO-UMC.

Age Group	Single-Herb Products (n=)	Multi-Herb Products (n=)	Mixed-Multiple Products (n=)	Total (n=) and % *
0–10	103	41	330	474 (2.2%)
11–20	125	47	246	418 (1.9%)
21–30	240	108	530	878 (4.0%)
31–40	345	173	780	1298 (5.9%)
41–50	636	423	1309	2368 (10.8%)
51–60	669	868	2201	3738 (17.0%)
61–70	234	796	2557	3587 (16.3%)
71–80	143	572	2278	2993 (13.6%)
81–90	46	193	933	1172 (5.3%)
91–100	12	16	116	144 (0.7%)
Unknown	1893	1418	1563	4874 (22.2%)
**Sex**				
Male	371	1016	5781	7168 (32.7%)
Female	3786	3510	6499	13,795 (62.9%)
Unknown	289	129	563	981 (4.5%)

* Calculated as the percentage of the sum of reported single-herb, multi-herb, and mixed-multiple products (n = 21,944).

**Table 12:** The most frequently reported plant species in single- and multi-herb and mixed-multiple products from reports submitted to the WHO-UMC.

Plant Species	Single-Herb Products (n=)	Multi-Herb Products (n=)	Mixed-Multiple Products (n=)	Total (n=) and % *
*Glycine max (L.)* Merr.	610	2920	12,139	15,669 (71.4%)
*Actaea racemosa* L.	1686	656	207	2549 (11.6%)
*Vitex agnus-castus* L.	1269	98	34	1401 (6.4%)
*Humulus lupulus* L.	23	657	172	852 (3.9%)
*Oenothera biennis* L.	535	5	81	621 (2.8%)
*Linum usitatissimum* L.	134	14	143	291 (1.3%)
*Trifolium pratense* L.	133	35	25	193 (0.9%)
*Pueraria montana* (Lour.) Merr.	16	157	10	183 (0.8%)
*Angelica sinensis* (Oliv.) Diels	37	112	32	181 (0.8%)
*Pueraria mirifica* Airy Shaw and Suvat.	3	1	1	4 (0.02%)

* Calculated as the percentage of the sum of reported single-herb, multi-herb, and mixed-multiple products (n = 21,944).

## Data Availability

The datasets for this manuscript are not publicly available because of the Lareb data protection policy. Requests to access the datasets should be directed to the second author and will be granted on reasonable request.

## Supplementary Materials

The following supporting information can be downloaded at: https://www.mdpi.com/article/10.3390/ph16081137/s1, Table S1: List of all ARs reported in single-herb products included in the scoping review; Table S2: List of all ARs reported in multi-herb products included in the scoping review; Table S3: Data on reports submitted to the WHO-UMC of which only single-herb products were used at the time the AR was reported or it was the only suspected product to cause the AR amongst other concomitant medication, categorized by System Organ Class (SOC) and the most commonly reported Preferred Terms (PTs); Table S4: Data on reports submitted to the WHO-UMC for single-herb products with multiple suspects categorized by System Organ Class (SOC) and the most commonly reported Preferred Terms (PTs); Table S5: Data on reports submitted to the WHO-UMC of which only multi-herb products were used at the time the AR was reported or it was the only suspected product to cause the AR amongst concomitant medication, categorized by System Organ Class (SOC) and the most commonly reported Preferred Terms (PTs); Table S6: Data on reports submitted to the WHO-UMC of which other products were concomitantly used with a multi-herb product which has also been classified as being suspect to cause an AR, categorized by System Organ Class (SOC) and the most commonly reported Preferred Terms (PTs); Table S7: Data on reports submitted to the WHO-UMC of which only mixed-multiple products were used at the time the AR was reported or it was the only suspected product to cause the AR amongst concomitant medication, categorized by System Organ Class (SOC) and the most commonly reported Preferred Terms (PTs); Table S8: Data on reports submitted to the WHO-UMC of which other products were concomitantly used with a mixed-multiple product which has also been classified as being suspect to cause an AR, categorized by System Organ Class (SOC) and the most commonly reported Preferred Terms (PTs).

## Abbreviations

ALAT	Alanin Aminotransferase
AR	Adverse Reaction
ER	Estrogen Receptor
GGT	Gamma-glutamyl transferase
HRT	Hormone-Replacement Therapy
ICSR	Individual Case Safety Report
LDL	Low-Density Lipoprotein
MedDRA	Medical Dictionary for Regulatory Activities
MeSH	Medical Subject Heading
PE	Phytoestrogen
PIDM	Program for International Drug Monitoring
PRISMA	Preferred Reporting Items for Systematic Reviews and Meta-Analyses
PT	Preferred Term
SERM	Selective Estrogen Receptor Modulator
SOC	System Organ Class
SSRI	Selective Serotonin Reuptake Inhibitor
UMC	Uppsala Minitoring Center
WHO	World Health Organization
