# Supplementary material for: Analysis of Safety Concerns on Herbal Products with Assumed Phytoestrogenic Activity
Source: Pharmaceuticals (Basel). 2023 Aug 10;16(8):1137. doi: 10.3390/ph16081137 (PMC10459077; doi:10.3390/ph16081137)
Supplement: Supplementary file 1 [file pharmaceuticals-16-01137-s001.zip › pharmaceuticals-2491826-supplementary.pdf]

Article-Supplementary Material

Analysis of Safety Concerns on Herbal Products with Assumed Phytoestrogenic Activity

A. Marije Tjeerdsma [1,2], Florence P. A. M. van Hunsel [2,3], Sonja van de Koppel [2], Corine Ekhart [2], Annabella Vitalone [4], and Herman J. Woerdenbag [1,*]

[1] Department of Pharmaceutical Technology and Biopharmacy, Groningen Research Institute of Pharmacy (GRIP), University of Groningen, Antonius Deusinglaan 1, 9713 AV Groningen, The Netherlands; marijetjeerdsma@gmail.com

[2] Netherlands Pharmacovigilance Centre Lareb, Goudsbloemvallei 7, 5237 MH 's-Hertogenbosch, The Netherlands; f.vanhunsel@lareb.nl (F.P.A.M.v.H.); vdkoppel@ziggo.nl (S.v.d.K.); c.ekhart@lareb.nl (C.E.)

[3] Department of PharmacoTherapy, -Epidemiology & -Economics, Groningen Research Institute of Pharmacy (GRIP), University of Groningen, Antonius Deusinglaan 1, 9713 AV Groningen, The Netherlands

[4] Department of Physiology and Pharmacology 'Vittorio Erspamer', Sapienza University of Rome, Piazzale Aldo Moro 5, 00185 Rome, Italy; annabella.vitalone@uniroma1.it

* Correspondence: h.j.woerdenbag@rug.nl; Tel.: +31-6-31921365

Abstract: Phytoestrogens (PEs) are plant-based compounds that can interact with estrogen receptors and are mainly used to treat menopausal complaints. However, the safety of products with assumed phytoestrogenic activity is not fully understood. This study aimed to identify plant species with assumed phytoestrogenic activity, review existing literature on their use and safety, and critically evaluate adverse reaction (AR) reports of single-herb, multi-herb, and mixed-multiple products, as submitted to the Netherlands Pharmacovigilance Centre Lareb and to VigiBase of the World Health Organization (WHO). In the Lareb database, the most commonly reported plant species to cause ARs (total of 67 reports) were *Actaea racemosa* L. (black cohosh) (47.8%), *Humulus lupulus* L. (hops) (32.8%), and *Glycine max* (L.) Merr. (soybean) (22.4%). In the VigiBase database (total of 21,944 reports), the top three consisted of *Glycine max* (L.) Merr. (71.4%), *Actaea racemosa* L. (11.6%), and *Vitex agnus-castus* L. (chaste tree) (6.4%). In the scoping review (total of 73 articles), *Actaea racemosa* L. (30.1%), *Glycine max* (L.) Merr. (28.8%), and *Trifolium pratense* L. (13.7%) were the most frequently mentioned plant species. ARs were most frequently reported in the system organ classes "gastrointestinal disorders", "skin and subcutaneous tissue disorders", "reproductive system and breast disorders", and "general disorders and administration site conditions". Furthermore, from the scoping review, it appeared that the use of products with assumed phytoestrogenic activity was associated with postmenopausal bleeding. It was concluded that, while the potential benefits of products with assumed phytoestrogenic activity have been extensively pursued, the potential occurrence of ARs after using these products is less well understood. This study highlights the need for further investigation and careful monitoring of these products to better understand their effects and ensure the safety and well-being of individuals using them.

Keywords: *Actaea racemosa* L.; drug-related side effects and adverse reactions; *Humulus lupulus* L.; Netherlands Pharmacovigilance Centre Lareb; *Glycine max* (L.) Merr.; phytovigilance; phytoestrogens; *Trifolium pretense* L.; VigiBase; *Vitex agnus-castus* L.; World Health Organization (WHO)

Table of Contents:

Table S1: List of all ARs reported in single-herb products included in the scoping review.
Table S2: List of all ARs reported in multi-herb products included in the scoping review.
Table S3: Data on reports submitted to the WHO-UMC of which only single-herb products were used at the time the AR was reported or it was the only suspected product to cause the AR amongst other concomitant medication, categorised by System Organ Class (SOC) and the most commonly reported Preferred Terms (PTs).
Table S4: Data on reports submitted to the WHO-UMC for single-herb products with multiple suspects categorised by System Organ Class (SOC) and the most commonly reported Preferred Terms (PTs).
Table S5: Data on reports submitted to the WHO-UMC of which only multi-herb products were used at the time the AR was reported or it was the only suspected product to cause the AR amongst concomitant

medication, categorised by System Organ Class (SOC) and the most commonly reported Preferred Terms (PTs).
Table S6: Data on reports submitted to the WHO-UMC of which other products were concomitantly used with a multi-herb product which has also been classified as being suspect to cause an AR, categorised by System Organ Class (SOC) and the most commonly reported Preferred Terms (PTs).
Table S7: Data on reports submitted to the WHO-UMC of which only mixed-multiple products were used at the time the AR was reported or it was the only suspected product to cause the AR amongst concomitant medication, categorised by System Organ Class (SOC) and the most commonly reported Preferred Terms (PTs).
Table S8: Data on reports submitted to the WHO-UMC of which other products were concomitantly used with a mixed-multiple product which has also been classified as being suspect to cause an AR, categorised by System Organ Class (SOC) and the most commonly reported Preferred Terms (PTs).

Table S1. List of all ARs reported in single-herb products included in the scoping review.

Plant Name with Synonyms (if Applicable)	Total AR	Reported AR	Number of Reports (n=)	Reported AR	Number of Reports (n=)
Actaea racemosa L. or *Cimicifuga racemosa* L. or *Cimicifuga foetida* L. (black cohosh)	615	Vaginal bleeding and spotting	107	Increased LDL	101
		Increased triglycerides	97	Increased total cholesterol	95
		Breast pain/enlargement	37	Gastrointestinal complaints/disorders*	24
		Proliferative endometrium	16	Breast/genital reproductive disorders*	16
		Musculoskeletal and connective tissue disorders*	15	Abdominal discomfort/pain	13
		Infections and infestations*	13	Oedema	8
		Nervous system disorders*	8	Leukorrhea	7
		Headache	7	Increased glucose	4
		Hypertension	4	Skin and subcutaneous tissue disorders*	3
		Constipation	2	Dyspeptic problems	2
		Allergic skin reactions	2	Cardiac disorders*	2
		Metabolism and nutrition disorders*	2	Other disorders*	2
		Psychiatric disorders*	2	Increased GGT	2
		Cervical carcinoma stage II	2	Injury, poisoning and procedural complications*	2
		Joint pain	1	Swollen feet and hands	1
		Phlebothrombosis	1	Blood and lymphatic disorders*	1
		General disorders*	1	Renal and urinary disorders*	1
		Cholelithiasis	1	Irritability	1

		Vascular disorders*	1	Central nervous disorders*	1
		Breast cancer	1	Vaginal candidiasis	1
		Coagulation activation	1	Weight gain	1
		Decreased HDL	1	Hypothyroidism	1
		Influenza	1	Tiredness	1
		Increased potassium	1	Depression	1
***Pueraria mirifica* Airy Shaw & Suvat. or *Pueraria candollei* var. *mirifica* (Airy Shaw & Suvat.) Niyomdham (*Pueraria mirifica* or Kwao Krua)**	115	Mastodynia	32	Dizziness	10
		Dyspepsia	9	Urticaria	9
		Vaginal spotting	8	Vaginal discharge	8
		Headache	6	Mild hypertension	3
		Body itching	3	Pelvic discomfort	2
		Myoma uteri development	2	Nausea	2
		Anemia	2	Constipation	2
		GI discomfort	1	Insomnia	1
		Numbness of lower extremities	1	Vaginal itching	1
		Vertigo	1	Urinary frequency	1
		Mild malaise + heavy head	1	Back pain	1
		Bitter taste	1	Bloating	1
		Drowsiness	1	Leg pain	1
		Benign breast cyst development	1	Chest discomfort	1
		Menorrhagia	1	Traumatic bone pain	1
		Palpitation	1		
***Glycine max* (L.) Merr (Soybean)**	88	Gastrointestinal disorders/complaints*	32	Unspecified	19
		Bleeding episodes/vaginal bleeding	10	Nausea and vomiting	8

		Headache	5	Breast pain	2
		Flatulence	2	Shortness of breath	2
		Epigastralgia	1	Pelvic discomfort	2
		Precordial pain	1	Menstrual alterations	1
		Metrorrhagia	1	Reddening of the skin	1
		Burning of the skin	1		
***Trifolium pratense* L. (Red clover)**	78	Cold or upper respiratory tract infection	19	Myalgia	18
		Headache	10	Nausea	12
		Arthralgia	12	Diarrhea	5
		Gastrointestinal problems*	1	Dizziness	1
***Rheum rhaponticum* or *Rheum altaicum* Losinsk. (False rhubarb)**	13	Vaginal spotting	2	Hyperplastic endometrium	2
		Viral infection of the upper respiratory tract	1	Aggravation of dizziness	1
		Duodenal ulcer	1	Cervical dysplasia	1
		Cardiomyopathy	1	Weight gain	1
		Back pain	1	Secretory endometrium	1
		Intercostal neuralgia	1		
***Linum usitatissimum* (Flaxseed)**	10	Gastrointestinal complaints*	10		
***Humulus lupulus* L. (Hops)**	8	Vaginal haemorrhage	4	Mild and transient rhinitis	3
		Throat pain	1		
***Styphnolobium japonicum* (L.) (Sophorae fructus)**	8	Cystitis	8		
	4	Flushing	2	Palpitations	1

Panax ginseng **C.A. Mey. (Korean ginseng)**		Insomnia	1		
Trigonella foenum-graecum **L. (Fenugreek)**	2	Exacerbation of migraines	1	Indigestion/reflux	1
Foeniculum vulgare **Mill. (Fennel)**	2	Allergic reaction	1	Feeling of severe heat	1
Silybum marianum **(L.) Gaertn. (Milk thistle)**	2	Mild gastric pain	2		
Epimedium bravicornu **Maxim. or Herba epidemii or** ***Epimedium sagittatum*** **(Sieb. EtZucc.) Maxim. or** ***Epimedium pubescens*** **Maxim. or** ***Epimedium koreanum*** **(Horny goat weed)**	2	Diarrhea	2		
**Chamaemelum nobile (L.) All (Chamomile)**	2	Burning sensation	2		
Cornus mas **L. (Cornelian cherry)**	1	Gastrointestinal problems*	1		

Table S2. List of all ARs reported in multi-herb products included in the scoping review.

Multi-Herb Combination (with Synonyms if Applicable)	Reported AR	Number of Reports (n=)	Reported AR	Number of Reports (n=)
Glycine max **(L.) Merr (Soybean) +** ***Angelica sinensis*** **(Oliv.) Diels (Dong quai) +** ***Actaea racemosa*** **L. or** ***Cimicifuga racemosa*** **L. (black cohosh)**	Nausea	1	Pruritus	1
Linum usitatissimum **(Flaxseed) +** ***Silybum marianum*** **(L.) Gaertn. (Milk thistle)**	Intestinal bloating	1		
Glycine max **(L.) Merr (Soybean) +** ***Trifolium pratense*** **L. (Red clover) +** ***Actaea racemosa*** **L. or** ***Cimicifuga racemosa*** **L. (black cohosh) +** ***Vitex agnus-castus*** **L. (chaste-tree) +** ***Valeriana officinalis L.*** **(valerian)**	Flatulence	2	Nausea	1
Glycine max **(L.) Merr (Soybean) +** ***Linum usitatissimum*** **(Flaxseed)**	Breast tenderness	5	Nausea	3
	Abdominal distention	2	Headache	2
Curcuma longa **L. (Turmeric) +** ***Pygeum africanum*** **or** ***Prunus africana*** **(Hook.f.) Kalkman (African plum) +** ***Serenoa repens*** **(W. Bartram) Small (Saw palmetto) +** ***Ganoderma lucidum*** **(fungus) +** ***Polygonum cuspidatum*** **Willd. Ex Spreng. (Japanese knotweed) + resveratrol +** ***Panax ginseng*** **C.A. Mey. (Korean ginseng) + quercetin**	Venous thromboembolic events	2		
Angelica sinensis **(Oliv.) Diels (Dong quai) +** ***Glycyrrhiza glabra*** **L. (licorice) +** ***Vitex agnus-castus*** **L. (chaste-tree) +** ***Actaea racemosa*** **L. or** ***Cimicifuga racemosa*** **L. (black cohosh) +** ***Foeniculum vulgare*** **Mill. (Fennel) +** ***Helonias opulus*** **or**	Aggravation of asthma	1	Headache	1
	Depression	1	Gastrointestinal upset	1

Chamaelirium luteum **A. Gray (False unicorn)**				
Epimedium bravicornu **Maxim. Or Herba epidemii or** ***Epimedium sagittatum*** **(Sieb. et Zucc.) Maxim. or** ***Epimedium pubescens*** **Maxim. or** ***Epimedium koreanum*** **(Horny goat weed) +** ***Rehmannia glutinosa*** **(Gaertn.) DC. (Chinese foxglove) +** ***Dioscorea batatas*** **or** ***Dioscorea polystachya*** **Turcz. (Chinese yam) +** ***Cornus officinalis*** **Siebold & Zucc. (Japanese cornel dogwood) +** ***Cinnamomum cassia*** **(L.) J. Presl (Chinese cinnamon) +** ***Morinda officinalis*** **F.C.How (Indian mulberry) +** ***Drynaria fortunei*** **or** ***Drynaria roosii*** **Nakaike (Gu-sui-bu)**	Heartburn	7	Nausea	3
	Breast discomfort	3	Stomach discomfort	3
	Constipation	2	Hysterectomy	2
	Liver enzyme abnormality	1		
Glycine max **(L.) Merr (Soybean) +** ***Ginkgo biloba*** **L. (Maidenhair tree) +** ***Salvia officinalis*** **L. (Common garden sage) +** ***Salvia pratensis*** **L. (Meadow sage) +** ***Glycyrrhiza glabra*** **L. (licorice)**	Breast cancer	1		
Vigna radiata **(L.) R. Wilczek (Mung bean) +** ***Eucommia ulmoides*** **Oliv. (Hardy rubber tree)**	Respiratory, thoracic, and mediastinal disorders	18	Nervous system disorders	13
	Musculoskeletal and connective tissue disorders	10	Gastrointestinal disorders	10
	General disorders and administration site conditions	6	Infections and infestations	2
	Skin and subcutaneous tissue disorders	2	Surgical and medical procedures	2
	Reproductive system and breast disorders	1	Immune system disorders	2
	Ear and labyrinth disorders	1	Psychiatric disorders	1

**MF101, an herbal extract containing 22 herbs**	Thickened endometrium	21	Loose stools	17
	Vaginal bleeding or spotting	13	Axillary adenocarcinoma of the skin	1
	Idiopathic pancreatitis	1		
***Angelica sinensis* (Oliv.) Diels (Dong quai) + *Astragalus membranaceus* (Fisch.) Bge. or *Astragalus mongholicus* Bunge (Mongolian milkvetch)**	Constipation	1	Epigastric discomfort	1
	Hypercholesterolemia	1	Rectal bleeding	1
***Glycine max* (L.) Merr (Soybean) + *Humulus lupulus* L. (Hops)**	Laryngitis	1	Nausea	1
***Angelica sinensis* (Oliv.) Diels (Dong quai) + *Vitex agnus-castus* L. (chaste-tree) + *Actaea racemosa* L. or *Cimicifuga racemosa* L. (black cohosh) + *Glycyrrhiza glabra* L. (licorice) + *Leonurus cardiaca* L. (Motherwort)**	Endometrial cancer	1		
***Trifolium pratense* L. (Red clover) + *Pueraria montana* (Lour.) Merr. (Kudzu) + *Capsicum annuum* L. (Red pepper) + Salvia officinalis L. (Common garden sage) + *Glycyrrhiza glabra* L. (licorice) + *Actaea racemosa* L. or *Cimicifuga racemosa* L. (black cohosh) + *Turnera diffusa* Willd. Ex Schult. (Damiana) + *Morella cerifera* or *Myrica cerifera* L. (Southern wax myrtle) + *Zingiber officinale* Roscoe (Ginger) + *Rubus idaeus* L. (Red raspberry) + *Valeriana officinalis* L. (valerian)**	Pancreatitis	1		
***Trifolium pratense* L. (Red clover) + *Glycine max* (L.) Merr (Soybean)**	Gastric intolerance	1		

***Actaea racemosa* L. or *Cimicifuga racemosa* L. (black cohosh) + *Vitex agnus-castus* L. (chaste-tree) + *Angelica sinensis* (Oliv.) Diels (Dong quai) + *Helonias opulus* or *Chamaelirium luteum* A.Gray (False unicorn) + *Glycyrrhiza glabra* L. (licorice) + *Eleutherococcus senticosus* Maxim. (Siberian ginseng) + *Punica granatum* L. (Pomegranate) + *Medicago sativa* L. (Alfalfa)**	Abnormal bleeding	15	Endometrial cancer	1
***Rehmannia glutinosa* (Gaertn.) DC. (Chinese foxglove) + Cornus officinalis Siebold & Zucc. (Japanese cornel dogwood) + *Dioscorea opposite* or *Dioscorea oppositifolia* L. (Chinese yam) + *Poria cocos* (Poria mushroom) + Cortex Moutan or *Paeonia suffruticosa* Andrews (Tree peony) + *Alismatis orientalis* or *Alisma plantago-aquatica* subsp. *orientale* (Sam.) Sam. (European water-plantain) + *Anemarrhena asphodeloides* Bunge (Zhimu) + *Glycyrrhiza uralensis* Fisch. Ex DC. (Chinese liquorice) + *Astragalus mongholicus* Bunge (Mongolian astragalus) + *Actractylodes macrocephala* Koidz. (Bai zhu Largehead)**	Rash	1	Stomach pains	1
	Influenza	1	Abdominal bloating	1
	Bloody nipple exudate	1		
***Glycine max* (L.) Merr (Soybean) + *Oenothera biennis* L. (Evening primrose) + *Actaea racemosa* L. or *Cimicifuga racemosa* L. (black cohosh)**	Weight gain	1	Edema	1
***Eucommia ulmoides* Oliv. (Hardy rubber tree) + *Cullen corylifolium* (L.) Medik. or Fructus psoraleae (Bu Gu Zhi) + *Salvia miltiorrhiza* Bunge (Danshen)**	Rash	1	Mild hydrosalpinx	1
	Rise in ALT	1		

Table S3. Data on reports submitted to the WHO-UMC of which only single-herb products were used at the time the AR was reported or it was the only suspected product to cause the AR amongst other concomitant medication, categorised by System Organ Class (SOC) and the most commonly reported Preferred Terms (PTs).

Vitex agnus-castus **L.**					
**Skin and subcutaneous tissue disorders**	462	Acne	107	Rash	68
		Pruritus	68		
**Gastrointestinal disorders**	406	Nausea	90	Abdominal discomfort	57
		Abdominal pain	52		
**Reproductive system and breast disorders**	401	Menstruation delayed	82	Intermenstrual bleeding	52
		Menstruation irregular	50		
**Nervous system disorders**	244	Headache	99	Dizziness	68
		Somnolence	16		
**General disorders and administration site conditions**	199	Drug ineffective	23	Fatigue	22
		Generalised oedema	16		
**Psychiatric disorders**	123	Insomnia	17	Depression	13
		Anxiety	12		
**Investigations**	63	Weight decreased	27	Blood pressure increased	6
		Hepatic enzyme increased	4		
**Respiratory, thoracic, and mediastinal disorders**	59	Dyspnoea	25	Respiratory distress	5
		Pharyngeal swelling	4		
**Cardiac disorders**	39	Palpitations	20	Cardiovascular disorder	5
		Arrhythmia	3		
**Eye disorders**	32	Visual impairment	4	Eye swelling	4
		Dry eye	3		
**Injury, poisoning and procedural complications**	31	Off label use	17	Expired product administered	2

		Exposure during pregnancy	2		
**Musculoskeletal and connective tissue disorders**	31	Back pain	6	Pain in extremity	4
		Arthralgia	3		
**Vascular disorders**	27	Flushing	10	Hot flush	7
		Haemorrhage	2		
**Immune system disorders**	25	Hypersensitivity	20	Anaphylactic reaction	2
**Infections and infestations**	20	Pustule	3	Rhinitis	3
		Gingivitis	2		
**Metabolism and nutrition disorders**	14	Decreased appetite	6	Food craving	2
		Increased appetite	2		
**Neoplasms benign, malignant, and unspecified**	9	Breast neoplasm	2	Uterine leiomyoma	2
**Pregnancy, puerperium, and perinatal conditions**	7	Abortion	3	Abortion spontaneous	2
**Renal and urinary disorders**	7	Pollakiuria	2	Bladder pain	1
**Blood and lymphatic system disorders**	6	Anaemia	2	Thrombocytopenia	2
		Lymphadenopathy	2		
**Endocrine disorders**	5	Hyperprolactinaemia	2	Goitre	1
**Ear and labyrinth disorders**	3	Deafness	1	Tinnitus	1
		Vertigo	1		
**Hepatobiliary disorders**	3	Hepatitis	1	Hepatitis acute	1
		Hyperbilirubinemia	1		
**Social circumstances**	1	Impaired work ability	1		
Actaea racemosa L.					
**Gastrointestinal disorders**	678	Abdominal discomfort	193	Nausea	116
		Abdominal pain	66		
**Skin and subcutaneous tissue disorders**	449	Pruritus	106	Rash	89
		Urticaria	53		
	372	Drug ineffective	45	Face oedema	43

**General disorders and administration site conditions**		Fatigue	37		
**Nervous system disorders**	227	Headache	83	Dizziness	62
		Somnolence	10		
**Investigations**	187	Weight increased	46	Hepatic enzyme increased	24
		Liver function test abnormal	22		
**Reproductive system and breast disorders**	172	Breast pain	35	Postmenopausal haemorrhage	20
		Intermenstrual bleeding	15		
**Psychiatric disorders**	108	Insomnia	28	Sleep disorder	20
		Restlessness	11		
**Hepatobiliary disorders**	90	Jaundice	14	Liver injury	10
		Hepatic function abnormal	10		
**Cardiac disorders**	78	Palpitations	54	Tachycardia	8
		Bradycardia	3		
**Vascular disorders**	77	Hot flush	36	Flushing	18
		Hypertension	8		
**Musculoskeletal and connective tissue disorders**	60	Arthralgia	10	Pain in extremity	9
		Myalgia	8		
**Eye disorders**	51	Visual impairment	8	Eye swelling	6
		Swelling of eyelid	4		
**Respiratory, thoracic, and mediastinal disorders**	43	Dyspnoea	11	Cough	5
		Respiratory distress	3		
**Injury, poisoning and procedural complications**	34	Medication error	5	Intentional overdose	3
		Incorrect dose administered	3		
**Immune system disorders**	33	Hypersensitivity	31	Type II hypersensitivity	1

		Anaphylactic reaction	1		
**Renal and urinary disorders**	28	Chromaturia	11	Dysuria	3
		Micturition urgency	3		
**Metabolism and nutrition disorders**	25	Decreased appetite	12	Increased appetite	4
		Fluid retention	2		
**Infection and infestations**	22	Rash pustular	4	Mastitis	2
		Pustule	2		
**Neoplasms benign, malignant, and unspecified**	6	Genital neoplasm malignant female	2	Breast cancer female	1
**Blood and lymphatic system disorders**	6	Thrombocytopenia	2	Anaemia	1
		Hypoplastic anaemia	1		
**Ear and labyrinth disorders**	5	Vertigo	3	Tinnitus	2
**Surgical and medical procedures**	3	Therapy change	1	Liver transplant	1
		Oophorectomy bilateral	1		
**Social circumstances**	2	Impaired work ability	1	Loss of personal independence in daily activities	1
**Endocrine disorders**	1	Thyroid pain	1		
**Product issues**	1	Product substitution issue	1		
Trifolium pratense **L.**					
**Gastrointestinal disorders**	45	Abdominal discomfort	12	Nausea	8
		Vomiting	5		
**Skin and subcutaneous tissue disorders**	35	Rash	8	Pruritus	7
		Urticaria	6		
**Nervous system disorders**	29	Headache	12	Dizziness	10
**General disorders and administration site conditions**	22	Drug ineffective	4	Malaise	2
		Fatigue	2		
	12	Myalgia	3	Arthralgia	3

**Musculoskeletal and connective tissue disorders**		Back pain	2		
**Reproductive system and breast disorders**	11	Heavy menstrual bleeding	5	Menstrual disorder	2
**Vascular disorders**	7	Hot flush	4	Hypertension	1
**Respiratory, thoracic, and mediastinal disorders**	6	Bronchospasm	2	Epistaxis	1
**Renal and urinary disorders**	4	Pollakiuria	1	Bladder pain	1
**Cardiac disorders**	3	Palpitations	2	Tachycardia	1
**Eye disorders**	3	Eye disorder	1	Dry eye	1
		Ocular hyperaemia	1		
**Infections and infestations**	3	Pharyngitis	1	Rhinitis	1
		Sinusitis	1		
**Psychiatric disorders**	3	Insomnia	2	Nightmare	1
**Investigations**	2	Liver function test increased	1	Blood creatine phosphokinase increased	1
**Congenital, familial, and genetic disorders**	1	Porphyria	1		
**Ear and labyrinth disorders**	1	Vertigo	1		
**Hepatobiliary disorders**	1	Hepatitis	1		
**Immune system disorders**	1	Hypersensitivity	1		
**Metabolism and nutrition disorders**	1	Hypocalcaemia	1		
**Neoplasms benign, malignant, and unspecified**	1	Endometrial cancer	1		
***Glycine max* (L.) Merr.**					
**General disorders and administration site conditions**	119	Pyrexia	33	Chest pain	17
		Chills	9		
**Skin and subcutaneous tissue disorders**	114	Rash	28	Urticaria	21
		Pruritus	16		
**Gastrointestinal disorders**	72	Nausea	19	Vomiting	15
		Abdominal pain	8		

**Infections and infestations**	62	Sepsis	8	Device related infection	8
		Fungal infection	8		
**Respiratory, thoracic, and mediastinal disorders**	54	Dyspnoea	20	Hypoxia	4
		Respiratory disorder	4		
**Investigations**	50	Liver function test abnormal	9	Blood culture positive	4
		Blood bilirubin increased	4		
**Nervous system disorders**	47	Headache	9	Dizziness	8
		Paraesthesia	7		
**Injury, poisoning and procedural complications**	45	Medication error	8	Overdose	6
		Infusion related reaction	3		
**Product issues**	36	Product odour abnormal	5	Product complaint	4
		Product quality issue	4		
**Vascular disorders**	31	Vasodilatation	9	Hypotension	5
		Haemorrhage	4		
**Hepatobiliary disorders**	27	Cholestasis	8	Hepatic failure	6
		Hepatic function abnormal	5		
**Metabolism and nutrition disorders**	20	Hypertriglyceridemia	6	Hyperlipidaemia	4
		Fluid retention	2		
**Musculoskeletal and connective tissue disorders**	20	Back pain	9	Pain in extremity	3
		Myalgia	2		
**Immune system disorders**	19	Hypersensitivity	8	Anaphylactic reaction	4
		Anaphylactic shock	2		
**Cardiac disorders**	13	Tachycardia	4	Palpitations	3
**Psychiatric disorders**	9	Anxiety	3	Nervousness	3
		Insomnia	2		

**Blood and lymphatic system disorders**	8	Disseminated intravascular coagulation	3	Blood disorder	1
**Renal and urinary disorders**	8	Chromaturia	2	Renal failure	2
**Eye disorders**	7	Eye pain	1	Conjunctival irritation	1
**Reproductive system and breast disorders**	7	Intermenstrual bleeding	2	Breast pain	1
**Surgical and medical procedures**	5	Liver transplant	2	Colectomy	1
**Ear and labyrinth disorders**	3	Vertigo	2	Tinnitus	1
**Pregnancy, puerperium, and perinatal conditions**	2	Abortion	1	Neonatal disorder	1
**Neoplasms benign, malignant, and unspecified**	1	Endometrial cancer	1		
Oenothera biennis **L.**					
**Skin and subcutaneous tissue disorders**	101	Pruritus	19	Urticaria	14
		Rash	12		
**Gastrointestinal disorders**	98	Nausea	19	Diarrhoea	19
		Abdominal pain	15		
**Nervous system disorders**	84	Headache	30	Seizure	14
		Dizziness	7		
**General disorders and administration site conditions**	47	Malaise	6	Pain	5
		Face oedema	4		
**Psychiatric disorders**	25	Insomnia	3	Confusional state	3
		Aggression	3		
**Eye disorders**	15	Ocular hyperaemia	3	Diplopia	2
		Vision blurred	2		
**Reproductive system and breast disorders**	15	Breast pain	5	Amenorrhoea	2
		Vaginal haemorrhage	2		
**Investigations**	13	Weight increased	5	Coagulation time prolonged	2
**Musculoskeletal and connective tissue disorders**	12	Myalgia	4	Arthralgia	3

**Respiratory, thoracic, and mediastinal disorders**	11	Bronchospasm	4	Dyspnoea	3
**Vascular disorders**	11	Flushing	3	Hypertension	2
**Infections and infestations**	10	Acne pustular	2	Pharyngitis	1
**Hepatobiliary disorders**	6	Hepatic function abnormal	4	Hepatitis	1
		Liver injury	1		
**Metabolism and nutrition disorders**	6	Gout	1	Decreased appetite	1
**Renal and urinary disorders**	6	Pollakiuria	2	Chromaturia	1
**Blood and lymphatic system disorders**	4	Thrombocytopenia	1	Leukoocytosis	1
**Immune system disorders**	4	Hypersensitivity	2	Seasonal allergy	1
		Serum sickness	1		
**Pregnancy, puerperium, and perinatal conditions**	3	Abortion	1	Ectopic pregnancy	1
		Haemorrhage in pregnancy	1		
**Neoplasms benign, malignant, and unspecified**	2	Hepatic cancer	1	Hodgkin's disease	1
**Cardiac disorders**	1	Tachycardia	1		
**Congenital, familial, and genetic disorders**	1	Hypospadias	1		
**Ear and labyrinth disorders**	1	Vertigo	1		
**Injury, poisoning and procedural complications**	1	Contusion	1		
***Humulus lupulus* L.**					
**Nervous system disorders**	5	Dizziness	2	Headache	2
		Tremor	1		
**Gastrointestinal disorders**	2	Dry mouth	1	Dyspepsia	1
**General disorders and administration site conditions**	2	Malaise	1	Pyrexia	1
**Psychiatric disorders**	2	Anxiety	1	Insomnia	1

**Skin and subcutaneous tissue disorders**	2	Rash	1	Urticaria	1
**Investigations**	1	Liver function test abnormal	1		
**Ear and labyrinth disorders**	1	Ear discomfort	1		
**Blood and lymphatic system disorders**	1	Activated protein C resistance	1		
***Pueraria montana* (Lour.) Merr**					
**Skin and subcutaneous tissue disorders**	1	Rash	1		
***Angelica sinensis* (Oliv.) Diels**					
**Reproductive system and breast disorders**	5	Amenorrhoea	2	Fibrocystis breast disease	1
		Breast pain	1	Mensturation irregular	1
**Skin and subcutaneous tissue disorders**	4	Pruritus	1	Rash	1
		Urticaria	1	Skin exfoliation	1
**Nervous system disorders**	4	Aphasia	1	Loss of consciousness	1
		Neurological decompensation	1	Psychomotor hyperactivity	1
**General disorders and administration site conditions**	3	Drug ineffective	1	Oedema peripheral	1
		Pyrexia	1		
**Hepatobiliary disorders**	1	Jaundice cholestatic	1		
***Linum usitatissimum* L.**					
**Gastrointestinal disorders**	7	Abdominal pain	2	Constipation	1
**Nervous system disorders**	6	Headache	3	Migraine	1
**Skin and subcutaneous tissue disorders**	4	Pain of skin	1	Rash	1
		Purpura	1	Skin irritation	1
**General disorders and administration site conditions**	4	Application site erythema	1	Application site pain	1
		Drug ineffective	1	Fatigue	1
**Ear and labyrinth disorders**	3	Vertigo	3		
**Metabolism and nutrition disorders**	3	Decreased appetite	2	Hypercholesterolaemia	1

**Psychiatric disorders**	2	Behaviour disorder	1	Hallucination	1
**Respiratory, thoracic, and mediastinal disorders**	2	Cough	1	Respiratory disorder	1
**Vascular disorders**	2	Flushing	1	Hypotension	1
**Reproductive system and breast disorders**	2	Heavy menstrual bleeding	1	Vaginal haemorrhage	1
**Eye disorders**	2	Mydriasis	1	Photopsia	1
**Cardiac disorders**	2	Atrioventricular block complete	1	Bradycardia	1
**Blood and lymphatic system disorders**	1	Anaemia	1		
**Endocrine disorders**	1	Thyroiditis	1		
**Hepatobiliary disorders**	1	Jaundice cholestatic	1		
**Investigations**	1	Prothrombin time prolonged	1		
**Pregnancy, puerperium, and perinatal conditions**	1	Premature labour	1		
***Pueraria mirifica* Airy Shaw & Suvat.**					
**Gastrointestinal disorders**	2	Nausea	1	Vomiting	1
**Nervous system disorders**	1	Dizziness	1		
**Skin and subcutaneous tissue disorders**	1	Fixed eruption	1		
**Vascular disorders**	1	Deep vein thrombosis	1		

Table S4. Data on reports submitted to the WHO-UMC for single-herb products with multiple suspects categorised by System Organ Class (SOC) and the most commonly reported Preferred Terms (PTs).

Vitex agnus-castus **L.**					
**Nervous system disorders**	21	Dizziness	5	Headache	4
		Tremor	2		
**Gastrointestinal disorders**	19	Nausea	6	Diarrhoea	3
		Abdominal pain	3		
**General disorders and administration site conditions**	17	Drug interaction	5	Condition aggravated	2
		Chills	1		
**Psychiatric disorders**	13	Depression	2	Insomnia	2
		Tearfulness	1		
**Skin and subcutaneous tissue disorders**	16	Pruritus	4	Angioedema	1
		Macule	1		
**Injury, poisoning and procedural complications**	7	Exposure during pregnancy	2	Intentional overdose	1
		Toxicity to various agents	1		
**Investigations**	7	Blood pressure increased	2	Heart rate increased	1
		Coagulation factor increased	1		
**Eye disorders**	6	Diplopia	1	Eye disorder	1
		Eye irritation	1		
**Hepatobiliary disorders**	4	Hepatitis	2	Drug-induced liver injury	1
		Jaundice	1		
**Reproductive system and breast disorders**	3	Polymenorrhoea	1	Hypomenorrhoea	1
		Breast discomfort	1		
**Cardiac disorders**	3	Palpitations	3		
**Immune system disorders**	2	Hypersensitivity	2		
**Infections and infestations**	2	Pustule	1	Rash pustular	1

**Pregnancy, puerperium, and perinatal conditions**	2	Unintended pregnancy	2		
**Respiratory, thoracic, and mediastinal conditions**	2	Dyspnoea	1	Throat irritation	1
**Congenital, familial, and genetic disorders**	1	Limb reduction defect	1		
**Metabolism and nutrition disorders**	1	Decreased appetite	1		
**Musculoskeletal and connective tissue disorders**	1	Back pain	1		
**Vascular disorders**	1	Deep vein thrombosis	1		
Actaea racemosa **L.**					
**General disorders and administration site conditions**	69	Drug interaction	10	Fatigue	10
		Drug ineffective	7		
**Investigations**	61	Hepatic enzyme increased	7	Blood alkaline phosphatase increased	4
		Weight increased	4		
**Hepatobiliary disorders**	60	Hepatitis	11	Jaundice	10
		Hepatocellular injury	5		
**Gastrointestinal disorders**	47	Nausea	15	Abdominal pain	4
		Diarrhoea	3		
**Skin and subcutaneous tissue disorders**	43	Rash	8	Pruritus	7
		Hyperhidrosis	4		
**Nervous system disorders**	39	Dizziness	8	Headache	5
		Serotonin syndrome	3		
**Psychiatric disorders**	22	Suicide attempt	3	Sleep disorder	3
		Insomnia	2		
**Reproductive system and breast disorders**	19	Heavy menstrual bleeding	2	Breast discomfort	1
		Endometriosis	1		
**Vascular disorders**	17	Hot flush	3	Hypotension	3
		Hypertension	2		

**Metabolism and nutrition disorders**	14	Decreased appetite	5	Hypokalaemia	3
		Type 2 diabetes mellitus	2		
**Musculoskeletal and connective tissue disorders**	12	Back pain	4	Arthralgia	1
		Myalgia	1		
**Respiratory, thoracic, and mediastinal disorders**	12	Dyspnoea	6	Cough	1
**Renal and urinary disorders**	11	Pollakiuria	2	Tubulointerstitial nephritis	2
**Cardiac disorders**	10	Palpitations	4	Tachycardia	1
**Eye disorders**	7	Photophobia	2	Vision blurred	1
**Immune system disorders**	7	Drug hypersensitivity	5	Food allergy	1
		Type I hypersensitivity	1		
**Blood and lymphatic system disorders**	4	Coagulopathy	2	Lymphopenia	1
		Thrombotic thrombocytopenic purpura	1		
**Infections and infestations**	4	Oral candidiasis	1	Rash pustular	1
		Urinary tract infection	1	Vaginal infection	1
**Injury, poisoning and procedural complications**	4	Incorrect route of product administration	1	Intentional overdose	1
		Overdose	1	Toxicity to various agents	1
**Neoplasms benign, malignant, and unspecified**	4	Breast cancer	1	Hormone receptor positive breast cancer	1
		Meningioma	1	Renal cancer	1
**Ear and labyrinth disorders**	2	Tinnitus	2		
**Endocrine disorders**	2	Hyperthyroidism	1	Thyrotoxic crisis	1
**Surgical and medical procedures**	2	Cancer surgery	1	Liver transplant	1
**Pregnancy, puerperium, and perinatal conditions**	1	Foetal death	1		

**Social circumstances**	1	Refusal of treatment by patient	1		
Trifolium pratense **L.**					
**General disorders and administration site conditions**	9	Adverse drug reaction	1	Fatigue	1
		Pyrexia	1		
**Gastrointestinal disorders**	6	Melaena	2	Abdominal pain upper	1
		Vomiting	2		
**Eye disorders**	4	Eye disorder	1	Miosis	1
		Vitreoretinal traction syndrome	1	Vitreous detachment	1
**Investigations**	4	Blood bilirubin increased	2	ALAT increased	1
		Blood lactate dehydrogenase increased	1		
**Nervous system disorders**	4	Headache	1	Neuroleptic malignant syndrome	1
		Syncope	1	Tremor	1
**Respiratory, thoracic, and mediastinal disorders**	4	Epistaxis	3	Pulmonary embolism	1
**Skin and subcutaneous tissue disorders**	4	Purpura	2	Erythema	1
		Rash erythematous	1		
**Reproductive system and breast disorders**	3	Postmenopausal haemorrhage	1	Vaginal cyst	1
		Vulval disorder	1		
**Injury, poisoning and procedural complications**	3	Nerve injury	1	Toxicity to various agents	1
		Off label use	1		
**Hepatobiliary disorders**	3	Acute hepatic failure	1	Hepatitis	1
		Jaundice	1		
**Blood and lymphatic system disorders**	2	Thrombocytopenia	1	Haemolytic anaemia	1

**Cardiac disorders**	2	Atrial fibrillation	1	Extrasystoles	1
**Metabolism and nutrition disorders**	1	Dehydration	1		
**Psychiatric disorders**	1	Anxiety	1		
**Vascular disorders**	1	Deep vein thrombosis	1		
Glycine max **(L.) Merr.**					
**Injury, poisoning and procedural complications**	139	Parenteral nutrition associated liver disease	50	Product packaging confusion	13
		Product dispensing error	11		
**General disorders and administration site conditions**	90	No adverse event	11	Death	9
		Pyrexia	7		
**Investigations**	60	Platelet count decreased	5	ASAT increased	3
		ALAT increased	3		
**Gastrointestinal disorders**	55	Vomiting	12	Nausea	8
		Diarrhoea	4		
**Metabolism and nutrition disorders**	52	Hyperglycaemia	10	Hyperlipidaemia	8
		Glucose tolerance impaired	7		
**Skin and subcutaneous tissue disorders**	49	Rash	8	Urticaria	8
		Pruritus	7		
**Nervous system disorders**	37	Seizure	5	Tremor	3
		Generalised tonic-clonic seizure	3		
**Respiratory, thoracic, and mediastinal disorders**	37	Dyspnoea	9	Hypoxia	3
		Pulmonary oedema	2		
**Hepatobiliary disorders**	36	Hepatic function abnormal	9	Hepatic failure	5
		Jaundice	4		
**Vascular disorders**	34	Shock	8	Hypotension	7
		Circulatory collapse	4		

**Cardiac disorders**	26	Tachycardia	5	Bradycardia	4
		Cardiac arrest	3		
**Immune system disorders**	17	Hypersensitivity	5	Anaphylactic shock	3
		Food allergy	2		
**Infections and infestations**	17	Sepsis	3	Adenovirus infection	2
**Blood and lymphatic system disorders**	15	Anaemia	3	Thrombocytopenia	3
		Pancytopenia	2		
**Psychiatric disorders**	14	Anxiety	2	Mental status changes	2
		Suicide attempt	2		
**Eye disorders**	4	Eye swelling	1	Eye movement disorder	1
		Visual impairment	1		
**Renal and urinary disorders**	7	Acute kidney injury	4	Oliguria	2
		Renal disorder	1		
**Musculoskeletal and connective tissue disorders**	6	Muscle spasms	2	Myalgia	1
**Product issues**	4	Device malfunction	1	Product barcode issue	1
		Thrombosis in device	1	Product quality issue	1
**Endocrine disorders**	2	Adrenal suppression	1	Hypothyroidism	1
**Ear and labyrinth disorders**	1	Vertigo	1		
**Neoplasms benign, malignant, and unspecified**	1	Prostate cancer	1		
**Pregnancy, puerperium, and perinatal conditions**	1	Abortion spontaneous	1		
**Surgical and medical procedures**	1	Toe amputation	1		
Oenothera biennis L.					
**Gastrointestinal disorders**	74	Diarrhoea	13	Abdominal discomfort	11
		Nausea	9		

**Nervous system disorders**	63	Dizziness	17	Headache	13
		Somnolence	5		
**Skin and subcutaneous tissue disorders**	49	Pruritus	9	Rash	7
		Acne	4		
**General disorders and administration site conditions**	46	Fatigue	5	Drug interaction	4
		Face oedema	4		
**Psychiatric disorders**	22	Insomnia	6	Hallucination	3
		Agitation	3		
**Investigations**	18	Blood glucose increased	4	Weight increased	3
		Blood pressure increased	2		
**Reproductive system and breast disorders**	13	Heavy menstrual bleeding	4	Menstrual disorder	2
		Menstruation irregular	2		
**Metabolism and nutrition disorders**	12	Decreased appetite	6	Hypoglycaemia	2
		Increased appetite	2		
**Respiratory, thoracic, and mediastinal disorders**	12	Dyspnoea	4	Epistaxis	3
		Cough	2		
**Hepatobiliary disorders**	11	Hepatocellular injury	4	Jaundice	2
		Hepatitis	2		
**Musculoskeletal and connective tissue disorders**	10	Arthralgia	3	Muscular weakness	2
**Renal and urinary disorders**	9	Dysuria	3	Haematuria	2
**Injury, poisoning and procedural complications**	7	Contusion	2	Wound	1
**Cardiac disorders**	6	Palpitations	4	Tachycardia	1
		Bradycardia total	1		
**Eye disorders**	5	Dry eye	1	Scleritis	1
**Immune system disorders**	5	Anaphylactic reaction	1	Hypersensitivity	1
**Vascular disorders**	5	Hot flush	2	Hypertension	1

**Congenital, familial, and genetic disorders**	3	Cleft palate	1	Congenital musculoskeletal disorder	1
		Limb reduction defect	1		
**Infections and infestations**	3	Creutzfeldt-Jakob disease	1	Cystitis	1
		Urinary tract infection	1		
**Blood and lymphatic system disorders**	2	Pancytopenia	1	Thrombocytopenia	1
**Ear and labyrinth disorders**	1	Vertigo	1		
**Endocrine disorders**	1	Inappropriate antidiuretic hormone secretion	1		
**Pregnancy, puerperium, and perinatal conditions**	1	Foetal death	1		
**Product issues**	1	Product substitution issue	1		
**Surgical and medical procedures**	1	Surgery	1		
***Humulus lupulus* L.**					
**Injury, poisoning and procedural complications**	10	Intentional overdose	4	Product administration error	2
**Nervous system disorders**	10	Headache	1	Dizziness	1
		Generalised tonic-clonic seizure	1		
**General disorders and administration site conditions**	9	Drug interaction	2	Fatigue	2
**Hepatobiliary disorders**	7	Hepatitis	2	Jaundice	2
**Psychiatric disorders**	7	Suicide attempt	3	Insomnia	1
		Irritability	1		
**Skin and subcutaneous tissue disorders**	6	Blister	1	Erythema	1
		Skin exfoliation	1		
**Gastrointestinal disorders**	4	Vomiting	2	Nausea	1
		Diarrhoea	1		

**Investigations**	3	Body temperature increased	1	International normalised ratio increased	1
		Prothrombin level decreased	1		
**Vascular disorders**	2	Hypertension	1	Peripheral coldness	1
**Musculoskeletal and connective tissue disorders**	2	Pain in extremity	1	Rhabdomyolysis	1
**Infections and infestations**	2	Sepsis	1	Urinary tract infection	1
**Cardiac disorders**	1	Tachycardia	1		
**Immune system disorders**	1	Cross sensitivity reaction	1		
**Pregnancy, puerperium, and perinatal conditions**	1	Cephalhematoma	1		
**Reproductive system and breast disorders**	1	Postmenopausal haemorrhage	1		
**Social circumstances**	1	Bedridden			
***Pueraria montana* (Lour.) Merr**					
**Investigations**	7	ALAT increased	3	ASAT increased	3
		Blood bilirubin increased	1		
**Gastrointestinal disorders**	6	Diarrhoea	2	Gastrointestinal pain	1
		Constipation	1		
**Skin and subcutaneous tissue disorders**	5	Rash	1	Urticaria	1
		Pruritus	1		
**Renal and urinary disorders**	3	Chronic kidney disease	1	Renal impairment	1
		Haematuria	1		
**Cardiac disorders**	2	Tachycardia	1	Palpitations	1
**General disorders and administration site conditions**	2	Swelling	1	Chest pain	1
**Hepatobiliary disorders**	2	Jaundice	1	Hepatotoxicity	1

**Nervous system disorders**	2	Headache	2		
**Vascular disorders**	1	Hot flush	1		
**Psychiatric disorders**	1	Insomnia	1		
**Eye disorders**	1	Eye pain	1		
***Angelica sinensis* (Oliv.) Diels**					
**Nervous system disorders**	17	Headache	1	Balance disorder	1
		Burning sensation	1		
**General disorders and administration site conditions**	15	Pain	2	Pyrexia	1
		Unevaluable event	1		
**Gastrointestinal disorders**	14	Nausea	3	Abdominal pain	3
		Abdominal pain upper	2		
**Investigations**	11	ALAT increased	1	Blood bilirubin increased	1
		Blood cholesterol increased	1		
**Reproductive system and breast disorders**	5	Vaginal haemorrhage	2	Adnexa uteri pain	1
		Withdrawal bleed	1	Prostatitis	1
**Musculoskeletal and connective tissue disorders**	5	Back pain	1	Flank pain	1
		Myalgia	1		
**Injury, poisoning and procedural complications**	5	Exposure during pregnancy	1	Inappropriate schedule of product administration	1
		Metal poisoning	1		
**Hepatobiliary disorders**	5	Drug-induced liver injury	1	Hepatitis toxic	1
		Jaundice	1		
**Skin and subcutaneous tissue disorders**	4	Rash	2	Pruritus	1
		Angioedema	1		
**Cardiac disorders**	3	Bradycardia	1	Tachycardia	1
		Extrasystoles	1		

**Infections and infestations**	3	Creutzfeldt-Jakob disease	1	Encephalitis	1
		Infection	1		
**Product issues**	3	Product contamination	1	Product label issue	1
		Product quality issue	1		
**Vascular disorders**	2	Hypertension	1	Hypotension	1
**Renal and urinary disorders**	2	Bladder pain	1	Urinary retention	1
**Psychiatric disorders**	2	Confusional state	1	Sleep disorder	1
**Blood and lymphatic system disorders**	2	Coagulopathy	2		
**Congenital, familial, and genetic disorders**	1	Limb reduction defect	1		
**Metabolism and nutrition disorders**	1	Abnormal loss of weight	1		
**Respiratory, thoracic, and mediastinal disorders**	1	Dyspnoea	1		
***Linum usitatissimum* L.**					
**General disorders and administration site conditions**	55	Drug interaction	9	Fatigue	6
		Asthenia	4		
**Gastrointestinal disorders**	44	Diarrhoea	7	Vomiting	6
		Dyspepsia	5		
**Investigations**	34	Blood cholesterol increased	6	Weight decreased	2
		Heart rate increased	1		
**Nervous system disorders**	31	Somnolence	4	Dizziness	4
		Headache	3		
**Respiratory, thoracic, and mediastinal disorders**	19	Dyspnoea	7	Respiratory disorder	5
		Breast pain	1		
**Skin and subcutaneous tissue disorders**	16	Pruritus	3	Hyperhidrosis	2
**Eye disorders**	11	Visual impairment	2	Cataract	2

**Musculoskeletal and connective tissue disorders**	11	Muscle spasms	5	Myalgia	2
		Musculoskeletal stiffness	2		
**Hepatobiliary disorders**	10	Hepatitis	2	Hepatocellular injury	1
		Hepatic failure	1		
**Infections and infestations**	10	Pneumonia	2	Abscess	1
		Urinary tract infection	1		
**Metabolism and nutrition disorders**	8	Decreased appetite	1	Hypokalaemia	1
		Hyperglycaemia	1		
**Vascular disorders**	8	Hypertension	4	Thrombosis	2
		Hot flush	1		
**Injury, poisoning and procedural complications**	7	Contusion	2	Limb injury	1
		Medication error	1		
**Renal and urinary disorders**	7	Dysuria	2	Renal pain	1
		Renal impairment	1		
**Psychiatric disorders**	6	Agitation	1	Fear	1
		Insomnia	1		
**Cardiac disorders**	5	Tachycardia	1	Extrasystoles	1
		Cardiac failure	1		
**Blood and lymphatic system disorders**	4	Anaemia	2	Leukopenia	1
		Thrombocytopenia	1		
**Product issues**	4	Device breakage	1	Device kink	1
		Device occlusion	1	Product formulation issue	1
**Immune system disorders**	3	Drug hypersensitivity	3		
**Ear and labyrinth disorders**	2	Vertigo	2		
**Neoplasms benign, malignant, and unspecified**	2	Breast cancer	1	Prostate cancer	1
**Reproductive system and breast disorders**	1	Breast pain	1		

**Social circumstances**	1	Social problem	1		
**Surgical and medical procedures**	1	Stent placement	1		

Table S5. Data on reports submitted to the WHO-UMC of which only multi-herb products were used at the time the AR was reported or it was the only suspected product to cause the AR amongst concomitant medication, categorised by System Organ Class (SOC) and the most commonly reported Preferred Terms (PTs).

**Gastrointestinal disorders**	1647	Dyspepsia	339
		Nausea	289
		Diarrhoea	180
**Skin and subcutaneous tissue disorders**	647	Pruritus	203
		Urticaria	136
		Rash	134
**General disorders and administration site conditions**	600	Fatigue	62
		Drug ineffective	52
		Pain	50
**Nervous system disorders**	566	Headache	170
		Dizziness	164
		Somnolence	44
**Investigations**	236	Weight increased	37
		Blood pressure increased	29
		ALAT increased	14
**Musculoskeletal and connective tissue disorders**	222	Pain in extremity	38
		Back pain	33
		Arthralgia	26
**Psychiatric disorders**	209	Anxiety	19
		Nightmare	16
		Sleep disorder	14
**Infections and infestations**	174	Nasopharyngitis	75
		Upper respiratory tract infection	14
		Cystitis	13
**Respiratory, thoracic, and mediastinal disorders**	136	Dyspnoea	44
		Cough	19

		Epistaxis	8
**Injury, poisoning and procedural complications**	107	Off label use	8
		Intentional product misuse	7
		Product use in unapproved indication	7
**Cardiac disorders**	99	Palpitations	54
		Tachycardia	11
		Arrhythmia	9
**Hepatobiliary disorders**	91	Hepatic function abnormal	19
		Hepatitis	18
		Jaundice	17
**Renal and urinary disorders**	91	Chromaturia	18
		Pollakiuria	11
		Nocturia	7
**Reproductive system and breast disorders**	91	Breast pain	11
		Vaginal haemorrhage	10
		Heavy menstrual bleeding	7
**Eye disorders**	89	Visual impairment	18
		Vision blurred	14
		Eye pain	10
**Metabolism and nutrition disorders**	70	Decreased appetite	21
		Fluid retention	11
		Cachexia	8
**Vascular disorders**	70	Hypertension	13
		Flushing	12
		Hot flush	8
**Immune system disorders**	59	Hypersensitivity	47
		Anaphylactic reaction	4
		Anaphylactic shock	4
**Ear and labyrinth disorders**	23	Vertigo	7

		Tinnitus	6
		Ear pain	4
**Product issues**	16	Product quality issue	3
		Product taste abnormal	3
		Product physical issue	2
**Neoplasms benign, malignant, and unspecified**	14	Hepatic cancer	4
		Breast cancer	3
		Prostate cancer	1
**Blood and lymphatic system disorders**	12	Leukopenia	6
		Neutropenia	3
		Eosinophilia	1
**Endocrine disorders**	8	Hyperthyroidism	3
		Pseudoaldosteronism	2
		Hypoestrogenism	1
**Surgery and medical procedures**	4	Surgery	1
		Therapy interrupted	1
		Liver transplant	1
**Congenital, familial, and genetic disorders**	4	Dermoid cyst	1
		Hypospadias	1
		Ventricular septal defect	1
**Pregnancy, puerperium, and perinatal conditions**	3	Ectopic pregnancy	2
		Abortion	1

Table S6. Data on reports submitted to the WHO-UMC of which other products were concomitantly used with a multi-herb product which has also been classified as being suspect to cause an AR, categorised by System Organ Class (SOC) and the most commonly reported Preferred Terms (PTs).

**Gastrointestinal disorders**	638	Dyspepsia	132
		Nausea	101
		Vomiting	56
**Skin and subcutaneous tissue disorders**	384	Pruritus	108
		Rash	68
		Urticaria	50
**General disorders and administration site conditions**	347	Face oedema	47
		Swelling	27
		Fatigue	26
**Nervous system disorders**	323	Dizziness	113
		Somnolence	59
		Headache	40
**Psychiatric disorders**	128	Insomnia	36
		Suicide attempt	28
		Confusional state	7
**Hepatobiliary disorders**	118	Jaundice	21
		Drug-induced liver injury	12
		Liver injury	10
**Investigations**	107	Weight increased	11
		ASAT increased	10
		ALAT increased	9
**Injury, poisoning and procedural complications**	84	Intentional overdose	18
		Intentional product misuse	8
		Exposure during pregnancy	8
**Respiratory, thoracic, and mediastinal disorders**	75	Dyspnoea	23
		Cough	12

		Interstitial lung disease	6
**Musculoskeletal and connective tissue disorders**	75	Myalgia	12
		Rhabdomyolysis	9
		Muscle spasms	8
**Cardiac disorders**	63	Palpitations	26
		Tachycardia	11
		Bradycardia	4
**Vascular disorders**	57	Hot flush	16
		Hypertension	10
		Hypotension	7
**Renal and urinary disorders**	56	Acute kidney injury	8
		Chromaturia	8
		Renal failure	7
**Metabolism and nutrition disorders**	54	Decreased appetite	10
		Hypokalaemia	10
		Dehydration	7
**Eye disorders**	46	Orbital oedema	6
		Visual impairment	5
		Vision blurred	4
**Infections and infestations**	37	Sepsis	4
		Acinetobacter sepsis	2
		Infection	2
**Reproductive system and breast disorders**	31	Breast pain	4
		Adnexa uteri pain	3
		Heavy menstrual bleeding	3
**Blood and lymphatic system disorders**	27	Thrombocytopenia	9
		Anaemia	3
		Eosinophilia	3
**Immune system disorders**	12	Hypersensitivity	5
		Anaphylactic reaction	4

		Anaphylactic shock	2
**Endocrine disorders**	12	Pseudoaldosteronism	6
		Hyperthyroidism	2
		Goitre	1
**Neoplasms benign, malignant, and unspecified**	11	Invasive lobular breast carcinoma	1
		Adenocarcinoma gastric	1
		Oesophageal carcinoma	1
**Product issues**	11	Product quality issue	7
		Product contamination	2
		Product contamination microbial	1
**Ear and labyrinth disorders**	9	Vertigo	4
		Tinnitus	3
		Deafness transitory	1
**Congenital, familial, and genetic disorders**	8	Mosaicism	1
		Congenital ectopic bladder	1
		Patent ductus arteriosus	1
**Pregnancy, puerperium, and perinatal conditions**	7	Abortion spontaneous	2
		Abortion late	1
		Premature baby	1
**Surgery and medical procedures**	6	Therapy interrupted	1
		Cancer surgery	1
		Hospitalisation	1
**Social circumstances**	2	Walking aid user	1
		Patient uncooperative	1

Table S7. Data on reports submitted to the WHO-UMC of which only mixed-multiple products were used at the time the AR was reported or it was the only suspected product to cause the AR amongst concomitant medication, categorised by System Organ Class (SOC) and the most commonly reported Preferred Terms (PTs).

**General disorders and administration site conditions**	5173	Chills	1208
		Pyrexia	1031
		Chest pain	645
**Gastrointestinal disorders**	3424	Nausea	1536
		Vomiting	669
		Diarrhoea	373
**Skin and subcutaneous tissue disorders**	2209	Pruritus	712
		Rash	520
		Urticaria	232
**Nervous system disorders**	1182	Headache	403
		Dizziness	397
		Tremor	94
**Respiratory, thoracic, and mediastinal disorders**	968	Dyspnoea	570
		Tachypnoea	114
		Cough	51
**Cardiac disorders**	784	Palpitations	351
		Cardiac flutter	240
		Tachycardia	108
**Vascular disorders**	715	Flushing	110
		Vascular pain	72
		Cyanosis	50
**Investigations**	607	Blood pressure increased	90
		Hepatic enzyme increased	65
		Oxygen saturation decreased	33
**Injury, poisoning and procedural complications**	326	Incorrect route of product administration	23
		Medication error	20

		Intentional product use issue	19
**Psychiatric disorders**	309	Tic	35
		Insomnia	30
		Anxiety	26
**Musculoskeletal and connective tissue disorders**	269	Back pain	73
		Myalgia	56
		Pain in extremity	35
**Metabolism and nutrition disorders**	267	Hyperglycaemia	42
		Decreased appetite	29
		Hypokalaemia	17
**Immune system disorders**	222	Anaphylactoid reaction	81
		Anaphylactic shock	58
		Hypersensitivity	55
**Hepatobiliary disorders**	174	Hepatic function abnormal	51
		Cholestasis	27
		Hepatitis	15
**Infections and infestations**	168	Phlebitis	230
		Sepsis	19
		Pneumonia	18
**Blood and lymphatic system disorders**	116	Thrombocytopenia	35
		Neutropenia	22
		Anaemia	12
**Renal and urinary disorders**	75	Dysuria	23
		Liver disorder	8
		Liver injury	7
**Product issues**	55	Product quality issue	9
		Product colour issue	6
		Product odour abnormal	6
**Eye disorders**	47	Vision blurred	7
		Visual impairment	5

		Periorbital oedema	4
**Reproductive system and breast disorders**	42	Heavy menstrual bleeding	10
		Vaginal haemorrhage	9
		Pelvic pain	3
**Neoplasms benign, malignant, and unspecified**	31	Malignant neoplasm progression	6
		Neoplasm malignant	3
		Neoplasm progression	3
**Ear and labyrinth disorders**	21	Vertigo	11
		Tinnitus	6
**Endocrine disorders**	8	Hyperthyroidism	3
		Adrenal insufficiency	1
		Hypothyroidism	1
**Congenital, familial, and genetic disorders**	8	Vascular malformation	3
		Newborn persistent pulmonary hypertension	1
**Surgery and medical procedures**	5	Catheter management	1
		Platelet transfusion	1
		Parenteral nutrition	1
**Social circumstances**	4	Bedridden	1
		Contraindication to medical treatment	1
		Family stress	1
**Pregnancy, puerperium, and perinatal conditions**	1	Abortion	1

Table S8. Data on reports submitted to the WHO-UMC of which other products were concomitantly used with a mixed-multiple product which has also been classified as being suspect to cause an AR, categorised by System Organ Class (SOC) and the most commonly reported Preferred Terms (PTs).

**General disorders and administration site conditions**	1799	Pyrexia	331
		Chills	189
		General physical health deterioration	171
**Gastrointestinal disorders**	1465	Nausea	439
		Vomiting	287
		Diarrhoea	161
**Skin and subcutaneous tissue disorders**	560	Pruritus	146
		Rash	123
		Urticaria	79
**Investigations**	495	Weight decreased	105
		Hepatic enzyme increased	47
		Laboratory test abnormal	31
**Nervous system disorders**	392	Dizziness	87
		Headache	51
		Taste disorder	25
**Metabolism and nutrition disorders**	361	Decreased appetite	121
		Hypophagia	65
		Feeding disorder	45
**Respiratory, thoracic, and mediastinal disorders**	309	Dyspnoea	160
		Cough	16
		Tachypnoea	14
**Injury, poisoning and procedural complications**	222	Off label use	19
		Intentional overdose	17
		Product dispensing error	13
**Infections and infestations**	201	Vascular device infection	36
		Sepsis	20

		Pneumonia	15
**Musculoskeletal and connective tissue disorders**	197	Back pain	59
		Pain in extremity	33
		Arthralgia	25
**Psychiatric disorders**	165	Suicide attempt	17
		Insomnia	15
		Depression	10
**Hepatobiliary disorders**	164	Hepatic function abnormal	33
		Jaundice	30
		Cholestasis	26
**Vascular disorders**	148	Hypotension	18
		Flushing	17
		Hot flush	16
**Cardiac disorders**	123	Tachycardia	46
		Palpitations	27
		Cardiac flutter	11
**Blood and lymphatic system disorders**	89	Thrombocytopenia	34
		Leukopenia	18
		Anaemia	8
**Renal and urinary disorders**	57	Acute kidney injury	8
		Renal pain	7
		Renal impairment	6
**Immune system disorders**	49	Hypersensitivity	18
		Anaphylactic reaction	9
		Anaphylactic shock	8
**Eye disorders**	30	Visual impairment	4
		Ocular hyperaemia	3
		Vision blurred	3
**Neoplasms benign, malignant, and unspecified**	27	Malignant neoplasm progression	3

		Basal cell carcinoma	3
		Breast cancer female	2
**Product issues**	17	Product packaging confusion	13
		Device occlusion	4
		Product formulation issue	2
**Pregnancy, puerperium, and perinatal conditions**	9	Foetal death	2
		Foetal growth restriction	2
		Premature baby	1
**Reproductive system and breast disorders**	9	Breast pain	3
		Nipple pain	1
**Endocrine disorders**	5	Hyperthyroidism	2
		Inappropriate antidiuretic hormone secretion	1
		Glucocorticoid deficiency	1
**Social circumstances**	3	Immobile	1
		Physical disability	1
		Alcohol use	1
**Surgery and medical procedures**	2	Limb immobilisation	1
		Hospitalisation	1
**Ear and labyrinth disorders**	1	Hypoacusis	1
**Congenital, familial, and genetic disorders**	1	Fallot's tetralogy	1
